# Prognostic implications of TOR1B expression across cancer types: a focus on basal-like breast cancer and cellular adaptations to hypoxia

**DOI:** 10.1007/s00432-024-05794-3

**Published:** 2024-06-06

**Authors:** Yan Zhang, Zhongfu Cai, Wen Chen, Lei Ye, Xinquan Wu

**Affiliations:** 1 Department of Integrated Traditional Chinese and Western Medicine Oncology, The Second Affiliated Hospital of Fujian Medical University, Quanzhou, 362000 Fujian China; 2 Department of Oncology, Nanan Hospital, Nanan, 362300 Fujian China; 3 Department of Breast Surgery, Women and Children’s Hospital, School of Medicine, Xiamen University, 10 Zhenhai Road, Xiamen, 361003 Fujian China

**Keywords:** TOR1B, Basal-like breast cancer, Cancer prognosis, Metabolic pathways, Hypoxia, HIF-1α

## Abstract

The TOR1B gene is known to play a pivotal role in maintaining cellular homeostasis and responding to endoplasmic reticulum stress. However, its involvement in cancer remains relatively understudied. This study seeks to explore the prognostic implications of TOR1B across various cancers, with a specific focus on Basal-like Breast Cancer (BLBC) and its underlying cellular mechanisms. Through comprehensive analysis of data from TCGA, TARGET, GEO, and GTEx, we investigated TOR1B expression and its correlation with patient outcomes. Furthermore, in vitro experiments conducted on BLBC cell lines examined the impact of TOR1B modulation on cell viability, apoptosis, and metabolic activity under varying oxygen levels. Our statistical analysis encompassed differential expression analysis, survival analysis, and multivariate Cox regression. Our findings indicate that TOR1B is overexpressed in BLBC and other cancers, consistently correlating with poorer prognosis. Elevated TOR1B levels were significantly associated with reduced overall and disease-free survival in BLBC patients. In vitro experiments further revealed that TOR1B knockdown augmented apoptosis and influenced metabolic activity, particularly under hypoxic conditions, highlighting its potential role in cancer cell adaptation to stress. Overall, our study underscores the importance of TOR1B in cancer progression, particularly in BLBC, where it serves as a notable prognostic indicator. The interaction between TOR1B and metabolic pathways, as well as its regulation by HIF-1α, suggests its significance in adapting to hypoxia, thereby positioning TOR1B as a promising therapeutic target for aggressive breast cancer subtypes.

**Supplementary Information:**

The online version contains supplementary material available at 10.1007/s00432-024-05794-3.

## Introduction

The TOR1B gene, encoding the torsinB protein, plays a crucial role in cellular homeostasis and is particularly significant in the functioning of the endoplasmic reticulum (ER). This gene is part of the AAA+ (ATPases Associated with diverse cellular Activities) protein family, which is involved in various cellular processes, including protein folding, trafficking, and degradation within the ER (Rose et al. 2015). The importance of TOR1B in maintaining the ER's integrity is critical for cellular health, as disruptions in ER function can lead to stress responses that are implicated in numerous diseases, including neurodegenerative disorders and cancer (Zhao et al. 2016).

In the realm of tumor research, investigations into the correlation involving tor1b remain notably sparse. To date, there exists just one study pinpointing tor1b as a predictive factor for bone metastasis in breast cancer patients. That study delved into the relationship between tor1b expression and the occurrence of bone metastasis events among breast cancer patients, utilizing two public datasets, E-MTAB-365 and GSE2034, for analysis. The findings suggest that breast cancer patients exhibiting high tor1b expression are more prone to bone metastasis (Nguyen et al. 2023). However, the study did not delve further into the molecular mechanisms underlying this phenomenon.

Approximately 15% of all breast cancers are composed of Basal-like breast cancer (BLBC), characterized by a lack of expression of ER/PR/HER2 and the presence of basal cytokeratins and c-Kit (Badowska-Kozakiewicz and Budzik 2016). BLBC, classified as a highly lethal molecular form of breast cancer, exhibits a significant fatality rate primarily attributed to the swift progression of both local and distant metastasis. Unlike other types of breast cancer at the molecular level, BLBCs do not have any specific therapies that target them. Besides chemotherapy, radiation therapy is the most widely used systemic treatment. Therefore, new therapeutic targets are urgently needed for BLBC (Alexandrou et al. 2019)

HIF-1α, also known as hypoxia-inducible factor-1α, serves as the primary nuclear transcription factor responsible for the hypoxic reaction in cancer cells. It has the ability to control angiogenesis, glycolysis, erythropoiesis, and apoptosis of cancer cells when exposed to a hypoxic setting (Zhang et al. 2017; Duan et al. 2020; Jin et al. 2020; You et al. 2021). Several types of tumors overexpress HIF-1α (Shen et al. 2017; Wang et al. 2019; Wan et al. 2020; Sun et al. 2021). Poor clinical outcomes and resistance to radiotherapy and chemoendocrine therapy are linked to an elevated level of HIF-1α in cases of breast cancer (Generali et al. 2006; Jögi et al. 2019).

In this study, we embarked on a pan-cancer analysis of tor1b expression and its prognostic implications across various malignancies, drawing upon data from TCGA and GEO databases. A focal point of our investigation honed in on the prognostic impact of tor1b expression across different subtypes of breast cancer. Our findings revealed that elevated tor1b expression poses a risk factor specifically for patients with BLBC. Employing ssGSEA (single-sample gene set enrichment analysis), we further dissected the relationship between tor1b and metabolic pathways in BLBC, uncovering a negative correlation between tor1b expression and the oxidative phosphorylation pathway. Concurrently, co-expression analysis hinted at a positive correlation between tor1b and HIF-1α expression. We postulate that within the realm of BLBC, tor1b might serve as one of the downstream components in the metabolic reprogramming orchestrated by HIF-1α. Our conjecture finds support through cellular experiments conducted under both hypoxic and normoxic conditions, which validated certain aspects of our conclusions.

## Materials and methods

### Data collection

We downloaded mRNA data on the expression levels of TOR1B across pan-cancer and related clinical information of samples from both the TCGA and TARGET databases. The tumor site (T), lymph-node involvement (N), and distant metastatic (M) were diagnosed according to the American Joint Committee on Cancer (AJCC, 8th edition). The control normal tissue samples were derived from normal samples within the TCGA database and the GTEx database. Using the datasets (GSE22796, GSE134359, GSE58212, and GSE86374) from the GEO database to further validate the findings, the data, downloaded in MINiML format, was processed according to the annotation information from the GPL96 platform. This process involved mapping probe IDs to gene symbols, excluding probes corresponding to multiple genes, and averaging the values for multiple probes that map to the same gene. During survival and multivariate analyses, cases were excluded if they had follow-up times less than 30 days, missing or unclear tumor TNM status, missing or unclear disease stage, or missing or unclear patient age and gender information.

### Differential expression analysis

The dataset GSE22796 from the GEO database, comprising 16 BCLC tumor samples and 8 normal breast tissue samples, was utilized to validate the aforementioned aberrant expression of TOR1B in BCLC tumor tissues. We additionally downloaded and conducted a combined analysis of three other breast cancer datasets: GSE134359, GSE58212, and GSE86374, incorporating 60 BCLC subtype breast cancer samples and 37 normal breast tissue samples. First, the mRNA expression data underwent batch effect removal to standardize the data across different datasets. Following this preprocessing, a differential expression analysis of TOR1B between the two tissue types was performed. Using immunohistochemistry (IHC) images from the The Human Protein Atlas (HPA, https://www.proteinatlas.org/) database, we examined the expression of TOR1B protein in breast cancer and normal breast tissue.

### Survival analysis

Survival analysis was performed with the ‘survival’ R package (http://cran.r-project.org/web/packages/survival/index.html). A median value as a cutoff was used to divide patients into two groups (high or low) based on their TOR1B mRNA expression levels. To investigate the impact of TOR1B expression and clinical factors on patients' Disease-Free Survival (DFS) and Overall Survival (OS), we performed univariate and multivariate Cox regression analyses. The *P* value cutoff value was 0.05. Since the GSE25066 dataset does not contain OS data for patients, Disease and recurrence free survival (DRFS) data were used to assess prognosis.

### Metabolic pathway analysis

To explore why high TOR1B expression leads to decreased OS, DFS and DRFS rates in patients with BLBC, we utilized the TCGA database to study the relationship between TOR1B expression and metabolic pathways in BLBC patient samples. We collected gene sets included in relevant pathways (10.3390/cancers12071788) and calculated enrichment scores for each pathway in each sample using the ssGSEA algorithm. Spearman correlation analysis was then used to calculate the correlation between TOR1B expression and pathway scores.

### The co-expression analysis between TOR1B and HIF1A in BLBC samples

We discovered a negative correlation between TOR1B expression and the intracellular oxidative phosphorylation pathway, leading us to hypothesize that high TOR1B expression might enhance the hypoxia tolerance of BLBC tumor cells by reducing their reliance on the normal aerobic energy pathway. This adaptation could prevent cancer cells from dying due to a lack of energy metabolism. HIF1A is one of the most critical genes affecting tumor cell hypoxia tolerance. Therefore, we employed Spearman correlation analysis to calculate the correlation between the expression levels of TOR1B and HIF1A in 140 BLBC samples from the TCGA database.

### Cell culture and cell transfection

MDA-MB-231 cell line was acquired from Fenghui Biotechnology Company located in Hunan, China. The cells were cultured in RPMI-1640 medium (Meilune, Dalian, China) with the addition of 10% FBS (Newzerum, Christchurch, New Zealand), 100 U/mL penicillin, and 100 μg/mL streptomycin. The incubation took place at 37℃ in a CO_2_ incubator with 5% CO_2_ and 95% humidity for a duration of 12 h. Subsequently, the cells were divided into two groups and cultured under either normoxic or hypoxic conditions for 24 h. The normoxic group consisted of cells that were cultured under normoxic conditions, which included 21% O_2_, 5% CO_2_, and 74% N2. Cells in the hypoxia group were cultured in a hypoxic incubator with an oxygen concentration of 1%, carbon dioxide concentration of 5%, and nitrogen concentration of 94%. HIF-1α was the target of short hairpin RNA (shRNA) designed and synthesized by Anburui Co., Ltd. (located in China, Anburui). This company also provided scrambled control shRNA for the experiment. Supplementary Table S2 contains the sequences of all shRNAs. MDA-MB-231 cells were seeded in 6-well dishes with full media at a density of 4×104 cells per well. Zolgene Biotech (Fuzhou, China) provided both the HIF-1α shRNA vectors and the shRNA-negative control vector. Cells were transduced with lentivirus when they reached approximately 40% confluency. The multiplicity of infection (MOI) was set at 10 to ensure a sufficient number of viable cells for the experiments and to minimize cellular stress caused by transfection. Subsequently, a stably transfected cell line was established using antibiotic selection. Following a 12-h incubation period at a temperature of 37 °C, the cells were rinsed and gathered for subsequent investigations. To assess the knockdown levels of HIF-1α, cells were transfected with three distinct shRNAs (sh1, sh2, and sh3) that targeted HIF-1α. Included in the experiment were a blank group (blank) and a negative control group (shRNA_NC) as control samples.

### Western blot

Western blotting was employed to analyze the protein expression obtained from BLBC cells, and quantification was performed using a BCA Protein Quantitation kit (Thermo Fisher Scientific, MA, USA). Proteins (10 μg) were separated using 10% SDS-PAGE electrophoresis and subsequently transferred onto PVDF membranes. After being blocked in 5% BSA in TBS for 1 h, the membrane was incubated with the primary antibody overnight. In this study, the researchers used the following primary antibodies: anti-HIF-1α (BOSTER, Wuhan, China), anti-TOR1B (ProteinTech, Wuhan, China), and anti-β-actin (ProteinTech, Wuhan, China). After being washed three times with PBS, the membranes were then incubated at room temperature for a period of 1–2 h with the secondary antibody. Protein bands were detected using an ultra-sensitive chemiluminescence kit (Bioscience, Shanghai, China) and analyzed with ImageJ software. MDA-MB-231 cells were cultured under both normoxic and hypoxic conditions, and Western blot analysis was conducted to assess the expression levels of TOR1B and HIF-1α. In addition, our research examined how the reduction of HIF-1α affected the levels of TOR1B in MDA-MB-231 cells when exposed to different oxygen environments. Experiments were repeated three times.

### Quantitative real-time PCR (qRT-PCR)

RNA was extracted from total cells using RNAiso Plus reagent (TaKaRa, Dalian, China). Approximately 1 µg of RNA was used to generate cDNA with the NovoScript Plus All-in-one 1st Strand cDNA Synthesis SuperMix (gDNA Purge) from Novoprotein in Shanghai, China. The expanded RT product was obtained by utilizing NovoStart^®^ SYBR qPCR Supermix (Novoprotein, E090-01B), and the data was collected with the assistance of an iQ5 Real-Time PCR Detection System (Bio-Rad, Hercules, CA). The relative expression was analyzed using the Ct (2^−△△Ct^) method, with β-actin as the internal control. The qPCR primers utilized by us are listed in Supplementary Table S3. QPCR was conducted to assess the levels of TOR1B and HIF-1α expression in MDA-MB-231 cells under varying oxygen conditions. Additionally, the impact of HIF-1α knockdown on TOR1B expression in MDA-MB-231 cells was examined in both normoxic and hypoxic conditions. All qPCR reactions were repeated three times.

### Flow cytometry

The assays were grouped by intervention as follows: normoxia NC (negative control) group, hypoxia NC group, normoxia TOR1B knockdown group, hypoxia TOR1B knockdown group, normoxia blank (blank control) group, and hypoxia blank group. The blank control was transfected with free reagent, while TOR1B was also transfected. An Annexin V-FITC/PI apoptosis detection kit (Meilun Biotech, Dalian, China) was employed for the identification of apoptosis. Following the harvest and centrifugation at 500-1000×*g* for 5 min, the cells were then exposed to 195 µl of binding buffer and 5 µl of Annexin V-FITC in a dark environment for 10 min at room temperature. We utilized a FACSCalibur flow cytometer from BD Biosciences in CA, USA to examine the fluorescence of Annexin V-FITC and PI. The data was analyzed using CellQuest software from BD Biosciences, located in CA, USA. Cell apoptosis rate = early apoptosis rate + late apoptosis rate. Experiments were repeated three times. One-way ANOVA followed by an LSD or a Dunnett’s T3 *t* test was used for multiple pairwise comparisons of apoptotic rate.

### Statistical analysis

Each experiment was conducted three times independently. The mean ± SD was calculated using SPSS 23.0 software (SPSS Inc., Chicago, IL, USA) for data analysis. To assess the disparities among groups, various statistical tests including one-way ANOVA, two-way ANOVA, and Student's *t* tests were employed. A significance level of less than 0.05 was deemed significant. Correlation analysis was performed using R to calculate Spearman's correlation coefficient (ρ) between two groups and scatter plots were drawn; a *ρ* < 0.1 was considered a weak correlation, 0.1 ≤ *ρ* < 0.4 a moderate correlation, and *ρ* ≥ 0.4 a strong correlation, with *p*<0.05 indicating statistical significance.

For prognosis analysis, Kaplan–Meier curves were initially used, with *p* values and Hazard Ratios (HR) with 95% Confidence Intervals (CI) obtained through log-rank tests and univariate Cox regression. A *p* < 0.05 was considered statistically significant. Univariate and multivariate analyses were conducted using Cox regression analysis, and forest plots were used to display each variable (*p* value, HR, and 95% CI), with *p* < 0.05 deemed statistically significant. Based on the results of multivariate Cox proportional hazards analysis, nomograms were created using the "rms" package to predict the total recurrence rate.

## Result

### Aberrant expression of TOR1B in pan-cancer tissues and its differential expression across breast cancer subtypes

**Fig. 1:**
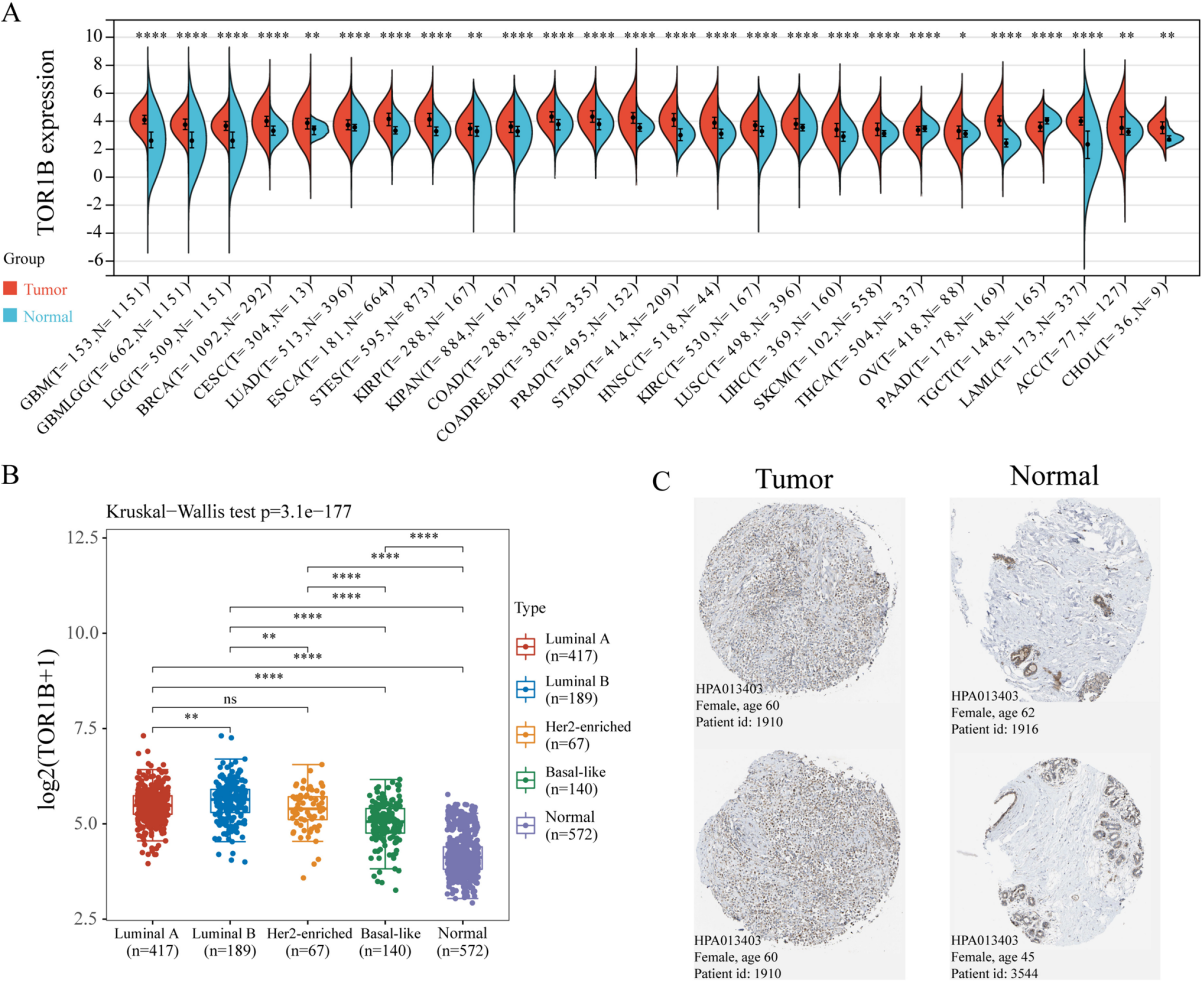
Aberrant expression of TOR1B in pan-cancer tissues and its differential expression across breast cancer subtypes. **A** The differential expression of TOR1B in pan-cancer tissues based on TCGA, TARGET, and GTEx databases. **B** Differential expression of TOR1B in breast cancer subtypes (PAM50) versus normal tissues from TCGA and GTEx databases. **C** IHC data from THPA database showed TOR1B was highly expressed in BC samples. *ACC* adrenocortical cancer, *BRCA* breast invasive carcinoma, *CESC* cervical and endocervical cancer, *CHOL* cholangiocarcinoma, *COAD* colon adenocarcinoma, *ESCA* esophageal carcinoma, *GBM* glioblastoma multiforme, *HNSC* head and neck squamous cell carcinoma, *KIRC* kidney clear cell carcinoma, *KIRP* kidney papillary cell carcinoma, *LAML* acute myeloid leukemia, *LGG* brain lower grade glioma, *LIHC* liver hepatocellular carcinoma, *LUAD* lung adenocarcinoma, *LUSC* lung squamous cell carcinoma, *OV* ovarian serous cystadenocarcinoma, *PAAD* pancreatic adenocarcinoma, *PRAD* prostate adenocarcinoma, *SKCM* skin cutaneous melanoma, *STAD* stomach adenocarcinoma, *TGCT* testicular germ cell tumor, *THCA* thyroid carcinoma, *STES* stomach and esophageal carcinoma, *GBMLGG* glioblastoma multiforme/lower grade glioma, *KIPAN* pankidney cohort, *COADREAD* colon adenocarcinoma/rectum adenocarcinoma esophageal carcinoma

The pan-cancer differential expression analysis of TOR1B, conducted with combined data from the TCGA, TARGET, and GTEx databases, indicates that TOR1B is differentially expressed in 26 types of cancer. It was found to have increased expression in 25 types of cancer tissues, including breast cancer, and exhibited decreased expression only in testicular germ cell tumors (TCGT) cancer tissues. Detailed information can be found in Figure 1A, Supplementary Material 1. Breast cancer tumor samples downloaded from TCGA were classified according to the PAM50 (50-gene set predictor using predication analysis of microarray method) typology into Luminal A (417 cases), Luminal B (189 cases), Her2-enriched (67 cases), and Basal-like (140 cases), with the Normal-like subtype having only 24 cases and therefore excluded from the analysis due to insufficient statistical power. Normal tissue samples, comprising adjacent non-cancerous tissues from TCGA and normal tissues from GTEx, totaled 572 samples and served as the control group. Following normalization of both tumor and normal tissue samples, differential expression analysis of TOR1B was performed. The results indicated that TOR1B expression was elevated in all four types of breast cancer compared to normal tissues. When comparing TOR1B expression among the four breast cancer subtypes, no significant difference was found between Luminal A and Her2-enriched subtypes, while differences in TOR1B expression levels were observed among the remaining subtypes, indicating significant variability in TOR1B expression across different breast cancer classifications (Fig. 1B, Supplementary Material 2). IHC staining pathological slides from the THPA database revealed that, compared to normal breast tissue, TOR1B protein expression was higher in breast cancer tissues (Fig. 1C).

### Impact of TOR1B expression on overall survival in various cancer types and breast cancer subtypes

**Fig. 2:**
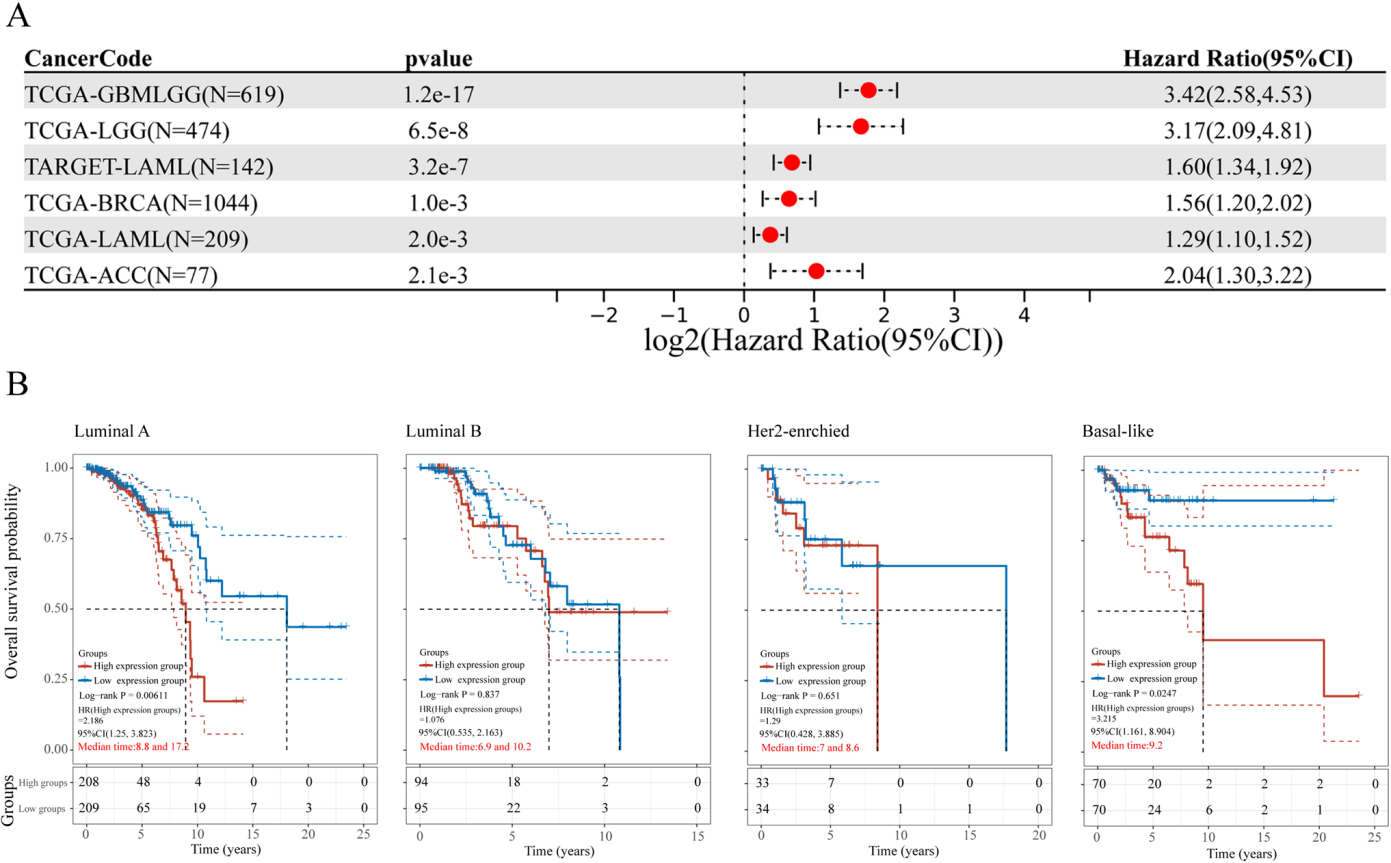
Impact of TOR1B expression on overall survival in various cancer types and breast cancer subtypes. **A** The Forest plot illustrates the significant impact of TOR1B on OS in six types of tumors from TCGA and TARGET databases, as determined by univariate Cox regression analysis. **B** The relationship between TOR1B expression and OS for the four breast cancer subtypes (PAM50)

We combined the expression data of TOR1B and follow-up information from 44 types of tumors in the TCGA and TARGET databases to analyze the relationship between TOR1B expression and the OS rate in these cancers. The results indicated that high expression of TOR1B is a risk factor affecting patient OS (HR > 1) in six types of tumors: Breast Invasive Carcinoma (BRCA), Glioblastoma Multiforme and Lower Grade Glioma (GBMLGG), Lower Grade Glioma (LGG), Acute Myeloid Leukemia (LAML), and Adrenocortical Carcinoma (ACC) (Fig. 2A, Supplementary Material 3). Further analysis of the relationship between TOR1B expression and OS for the four breast cancer subtypes revealed that high TOR1B expression is a risk factor affecting the OS of patients with Basal-like (HR=3.22, *P* < 0.05) and Luminal A (HR=2.19, *P* < 0.05) subtypes. However, there was no statistically significant association between TOR1B expression and the OS of patients with Luminal B and Her2-enriched subtypes (*P* > 0.05) (Fig. 2B, Supplementary Material 4).

### Further validation of differential expression and survival analysis using the GEO database

**Fig. 3:**
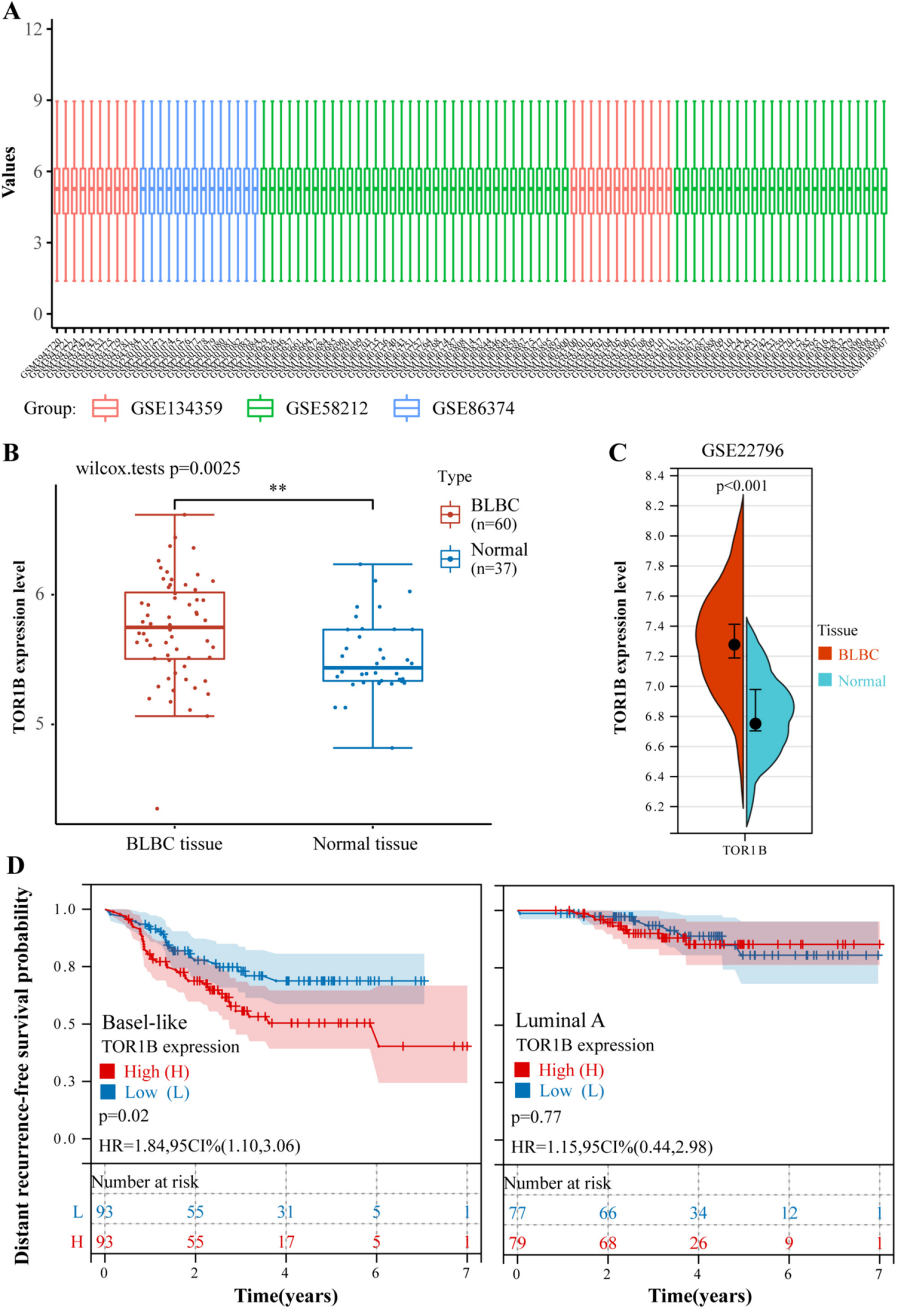
The GEO database was used to validate differentiating expression and survival analyses. **A** Boxplot after batch correction of three GEO datasets, with different colors representing different datasets. Rows indicate samples, and columns represent gene expression values within the samples. **B** Differential expression analysis using TOR1B expression data from BCLC tumor tissues and normal tissues across three GEO datasets indicates aberrantly high expression of TOR1B in BCLC tumor tissues. **C** Differential expression analysis of TOR1B expression data from BCLC tumor tissues and normal tissues in the GSE22796 dataset indicates aberrantly high expression of TOR1B in BCLC tumor tissues. **D** Impact of TOR1B expression on DRFS in Basal-like and Luminal A breast cancer patients from the GSE25066 dataset. High TOR1B expression correlates with reduced DRFS in Basal-like breast cancer (*P* < 0.05), with no significant impact on Luminal A DRFS (*P* > 0.05)

The integration of TOR1B expression data from tumor and normal tissues across three GEO datasets for differential expression analysis revealed that TOR1B was aberrantly highly expressed in BCLC tumor tissues (Fig. 3A, B, Supplementary Material 5). This finding was corroborated by a separate analysis of TOR1B expression data from BCLC tumor and normal tissues within the single dataset GSE22796, which also indicated abnormally high expression of TOR1B in BCLC tumor tissues (Fig. 3C, Supplementary Material 6). These outcomes were consistent with prior results obtained using the TCGA database, which had identified aberrant expression of TOR1B in BCLC tumor tissues. Analysis of the impact of TOR1B expression on DRFS in Basal-like and Luminal A breast cancer patients from the GSE25066 dataset showed that high TOR1B expression was associated with decreased DRFS in Basal-like breast cancer (*P* < 0.05) but had no significant effect on Luminal A DRFS (*P* > 0.05) (Fig. 3D, Supplementary Material 7). When these results were compared with previous findings from the TCGA database regarding the impact of TOR1B expression on OS rates in BCLC patients, it became evident that high TOR1B expression was a risk factor for poor prognosis in BCLC patients.

### Multivariate analysis of prognostic factors in patients with BCLC

In the univariate analysis, prognostic indicators that may affect OS include TOR1B expression levels (*P* < 0.05, HR = 4.308) and lymph node status (*P* < 0.05, HR = 4.196). Prognostic indicators that may affect DFS include TOR1B expression levels (*P* < 0.05, HR = 4.305), tumor size (*P* < 0.05, HR = 3.89), and metastasis (*P* < 0.05, HR = 22.417). In the multivariate analysis, prognostic indicators that may affect OS include TOR1B expression levels (*P* < 0.05, HR = 4.83) and tumor size (*P* < 0.05, HR = 6.178). Prognostic indicators that may affect DFS include TOR1B expression levels (*P* < 0.05, HR = 6.028), tumor size (*P* < 0.05, HR = 7.366), and metastasis (*P* < 0.05, HR = 16.942) (Table 1, Supplementary Material 8). TOR1B expression significantly impacts both OS and DFS, serving as a crucial prognostic indicator in BCLC outcomes.

**Table 1:** Univariate and multivariate Cox regression analyses for OS and DFS in 109 patients with BLBC

OS	Univariate analysis		Multivariate analysis	
Factors	*P* value	HR (95% CI)	*P* value	HR (95% CI)
TOR1B (high expression level vs. low expression level)^*^	0.027	4.308 (1.18–15.733)	0.034	4.83 (1.13–20.642)
Age (≥ 40 vs. < 40)	0.409	3.186 (0.012–46851.016)	0.985	1007554.91 (0.001–NA)
Tumor size (T3–4 vs. T1–2)^**^	0.075	3.318 (0.885–12.441)	0.017	6.178 (1.375–27.683)
Lymph node status (N1–3 vs. N0)^**^	0.016	4.196 (1.31–13.436)	0.054	3.33 (0.980–11.311)
Metastasis (M1 vs. M0)^**^	0.163	3.555 (0.598–21.125)	0.811	1.261 (0.188–8.448)
DFS				
Factors				
TOR1B (high expression level vs. low expression level)^*^	0.026	4.305 (1.179–15.724)	0.016	6.028 (1.394–26.066)
Age (≥ 40 vs. < 40)	0.607	1.711 (0.221–13.219)	0.704	1.526 (1.172–13.537)
Tumor size (T3–4 vs. T1–2)^**^	0.042	3.89 (1.051–14.398)	0.011	7.366 (1.571–34.532)
Lymph node status (N1–3 vs. N0)^**^	0.169	2.153 (0.722–6.419)	0.593	1.382 (0.421–4.534)
Metastasis (M1 vs. M0)^**^	0.008	22.417 (2.224–225.915)	0.022	16.942 (1.499–191.457)

^*^Based on the median value of TOR1B expression, patients were divided into low and high expression groups

^**^Tumor stage (T), lymph node metastasis stage (N) and Metastasis (M) were divided into two groups according to the 8th American Joint Committee on Cancer (AJCC) editions

### Metabolic pathway and co-expression analyses suggest a correlation between TOR1B and HIF1A in BLBC

**Fig. 4:**
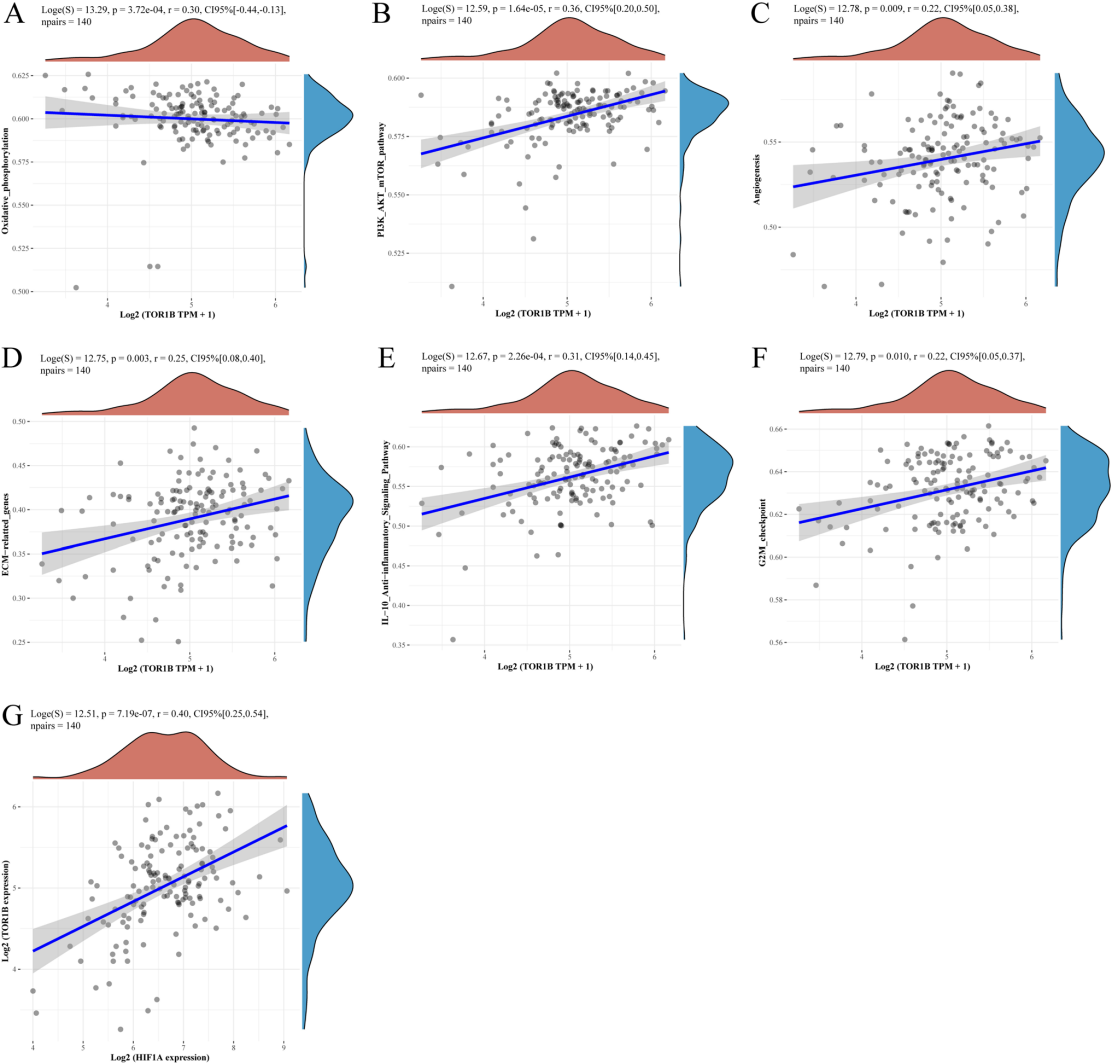
An analysis of metabolic pathways and co-expression suggested that TOR1B and HIF1A were correlated in BLBC. **A**–**F** Correlations between TOR1B Expression and Metabolic Pathways in BLBC: Negative correlation with oxidative phosphorylation (*ρ* = -0.3) and positive correlations with PIK3/AKT/mTOR (*ρ* = 0.36), angiogenesis (*ρ* = 0.22), ECM-related pathways (*ρ* = 0.22), IL-10 pathway (*ρ* = 0.31), and G2M checkpoint (*ρ* = 0.22); all significant (*p* < 0.05). **G** Positive Correlation Between HIF1A and TOR1B Expression in TCGA BLBC Samples Indicating Co-expression (*ρ* = 0.4, *p* < 0.01)

After conducting an analysis of TOR1B-related metabolic pathways using the ssGSEA algorithm, we found in BLBC samples that TOR1B expression is significantly negatively correlated with the intracellular oxidative phosphorylation pathway (*ρ*=−0.3, *p* < 0.01) and positively correlated with the PIK3/AKT/mTOR pathway (*ρ*=0.36, *p* < 0.01), angiogenesis (*ρ*=0.22, *p* < 0.01), ECM-related pathways (*ρ*=0.22, *p* < 0.01), IL-10 anti-inflammatory pathway (*ρ*=0.31, *p* < 0.01), and G2M checkpoint pathway (*ρ*=0.22, *p* < 0.05) (Fig. 4A–F, Supplementary Material 9). We hypothesized that high TOR1B expression, by reducing reliance on the normal aerobic energy pathway, may enhance the hypoxia tolerance of BCLC tumor cells, potentially preventing cell death due to energy deficiency. HIF1A, one of the most critical genes affecting tumor cell hypoxia tolerance, showed a positive correlation with TOR1B expression in BLBC samples from the TCGA database (*ρ*=0.4, *p* < 0.01), indicating co-expression between the two (Fig. 4G, Supplementary Material 10).

### The expression of HIF-1α and TOR1B in BLBC cells was further increased under hypoxic conditions

**Fig. 5:**
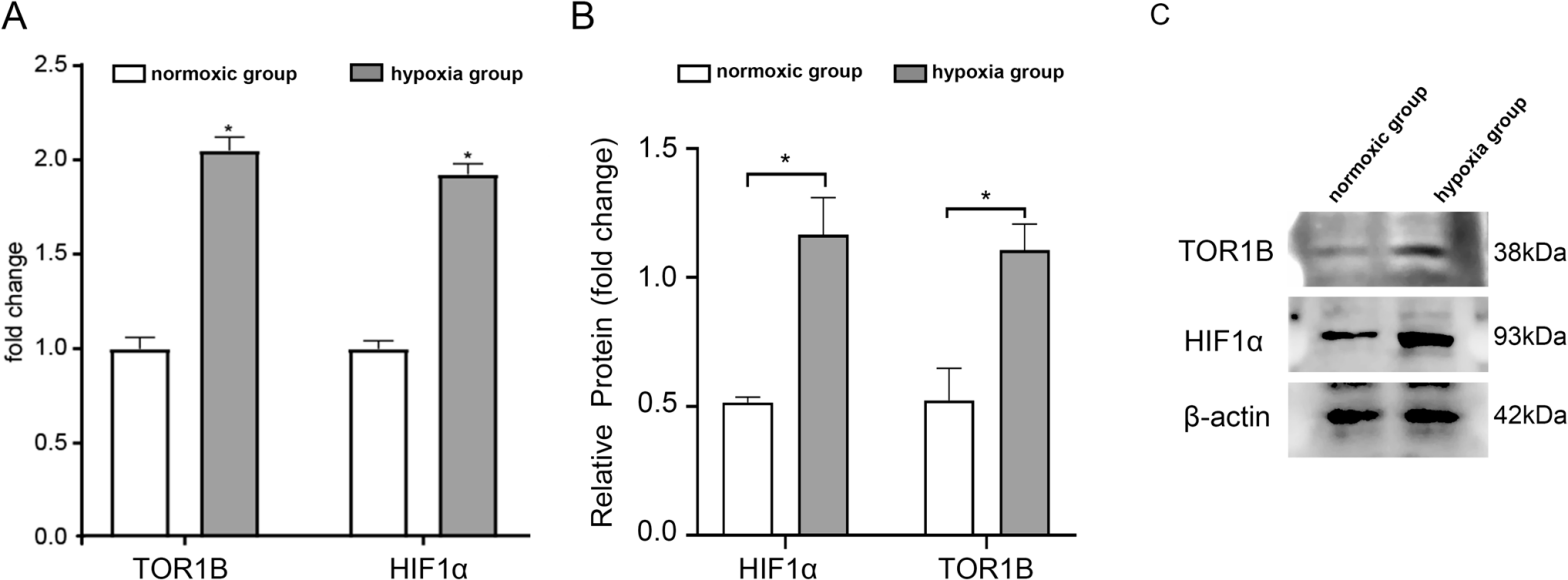
BLBC cells experience an increase in HIF-1α and TOR1B levels when exposed to hypoxia. **A** qRT-PCR analysis showed that hypoxia resulted in increased HIF-1α and TOR1B mRNA levels (*p < 0.05). **B**, **C** A significantly increased expression of HIF-1α and TOR1B were detected by western blotting following hypoxia induction (*p < 0.05)

In MDA-MB-231 cells, the mRNA and protein levels of HIF-1**α** and TOR1B were significantly increased by hypoxia induction, as shown by the qRT-PCR and western blot findings (Fig. 5A–C).

### HIF-1α induces TOR1B expression in BLBC cells

**Fig. 6:**
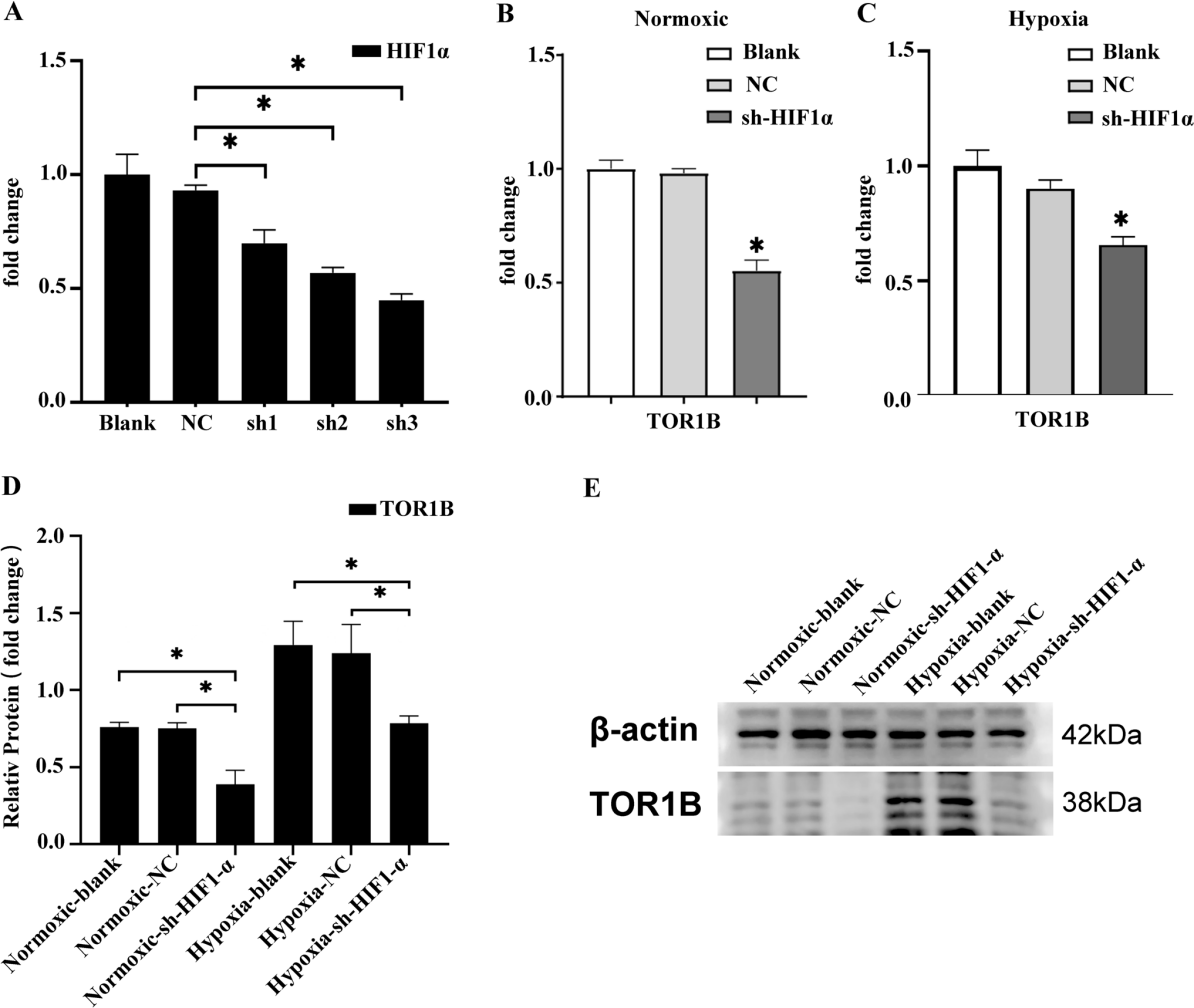
In BLBC cells, HIF-1α triggers the expression of TOR1B. Abbreviations: NC, negative control; Blank, blank control; sh, short hairpin RNA. **A** By RT‒qPCR, the HIF-1α knockdown efficiency in BLBC cells was measured. sh-HIF-1α (sh3) exhibited the best silencing effect and was used in subsequent experiments; *p < 0.05. The results of RT‒qPCR **B**, **C** and Western blot analyses **D**, **E** demonstrated that the suppression of HIF-1α led to a reduction in the expression level of TOR1B in BLBC cells. These observations were consistent for cells cultured under both normoxic and hypoxic conditions,*p < 0.05

We designed three shRNAs to silence HIF-1α. Experiments were performed using the sh-HIF-1α-3 (sh3) which exhibited the most effective interference outcomes (Fig. 6A). The results from RT-qPCR and Western blot analysis in BLBC (MDA-MB-231) cells demonstrated a significant decrease in TOR1B expression following the transfection of sh-HIF-1α-3. The results remained the same in cells cultured under both normal and low oxygen conditions (Fig. 6B–E). This finding indicates that HIF1a could potentially regulate the expression of TOR1B in BLBC cells.

### TOR1B mediates BLBC cell apoptosis

**Fig. 7:**
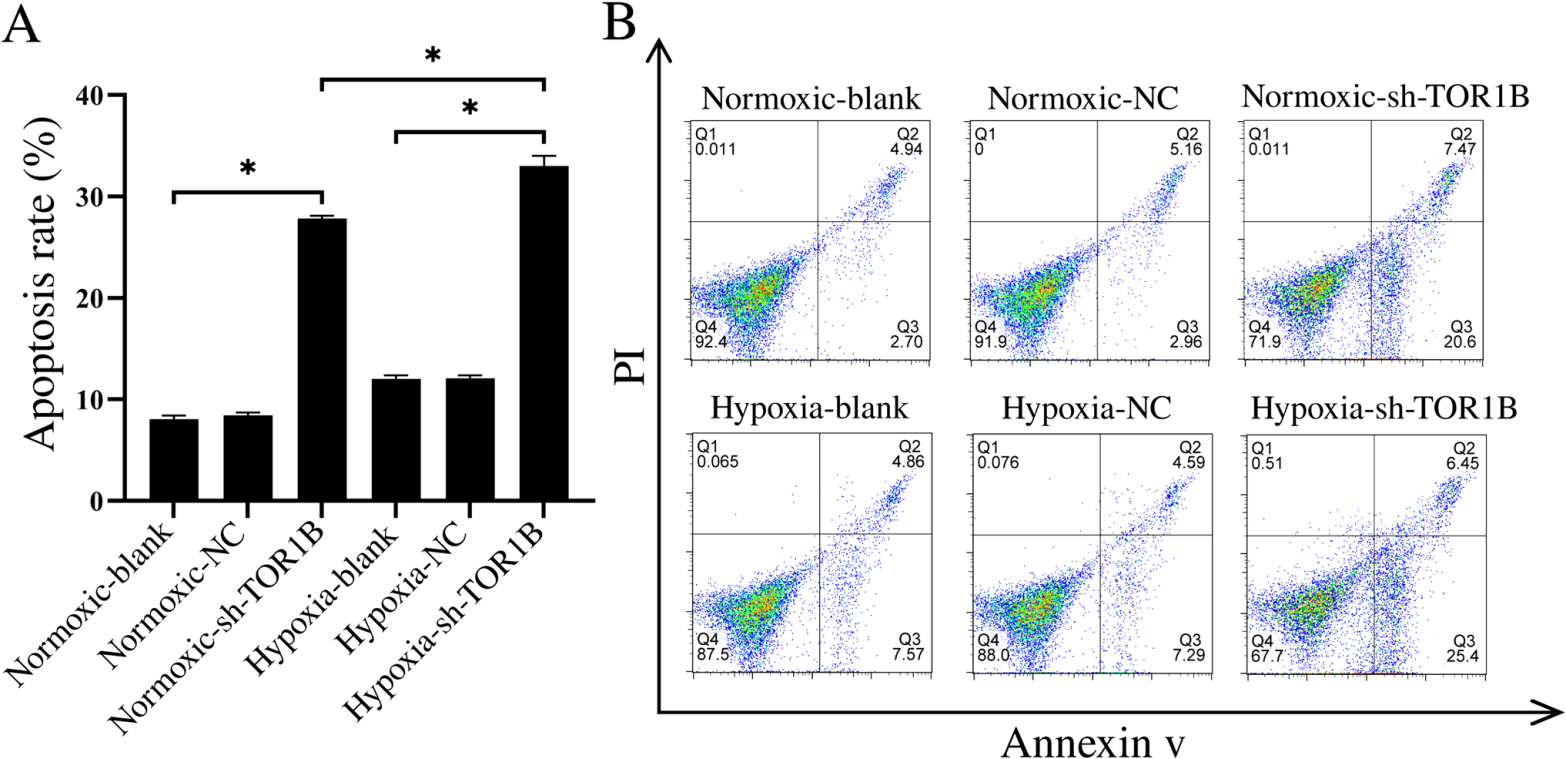
Flow cytometry results with Annexin V-FITC/PI staining. The cells were divided into groups according to treatment status as follows: normoxia NC (negative control) group, hypoxia NC group, normoxia TOR1B knockdown group, hypoxia TOR1B knockdown group, normoxia blank (blank control) group, and hypoxia blank group. **A** Results of multiple pairwise comparisons of apoptotic rate test showed a significant difference between all groups (*p < 0.001) but the normoxia blank group vs normoxia NC group and hypoxia blank group vs hypoxia NC group. **B** Quantification of viable (Q4: Annexin V-FITC−/PI−), early apoptotic (Q3: Annexin V-FITC + /PI−), late apoptotic (Q2: Annexin V-FITC + /PI +) and necrotic cells (Q1: Annexin V-FITC− /PI +) by AnnexinV-FITC/PI assay. A *P* value less than 0.05 was considered to indicate statistical significance

Results of multiple pairwise comparisons of apoptotic rate showed a significant difference between all groups (*p* < 0.001) but the normoxia blank group vs normoxia NC group (*p* = 0.371) and hypoxia blank group vs hypoxia NC group (*p* = 0.898). Overall, the rate of apoptosis under hypoxic conditions is higher than under normoxic conditions. Downregulating TOR1B increases the rate of apoptosis in both conditions, with this effect being more pronounced under hypoxia (Fig. 7A, B).

## Discussion

The TOR1B gene, also known as torsin family 1 member B, plays a pivotal role in the maintenance of cellular homeostasis and the endoplasmic reticulum's stress response. As a member of the AAA+ protein family, TOR1B is implicated in a variety of critical cellular processes including protein processing and trafficking within the ER, a key site for protein folding and quality control (Rose et al. 2015). The ER stress response, facilitated by TOR1B, is crucial for cellular adaptation to stress, ensuring that misfolded proteins are either correctly refolded or degraded to maintain cellular function (Zhao et al. 2016). Furthermore, TOR1B's role extends to the regulation of the nuclear envelope's integrity and dynamics, influencing processes such as nuclear division and chromatin organization (Luithle et al. 2020). Recent studies have shed light on the significant relationship between the TOR1B gene and cancer, specifically its role in breast cancer and its association with bone metastasis (BM). Research indicates that the expression level of TOR1B is significantly upregulated in breast cancer (BC) patients who develop bone metastasis (Nguyen et al. 2023). This upregulation has been validated across different patient datasets, highlighting TOR1B's potential role in the progression of breast cancer to more advanced, metastatic stages. In this study, we conducted a pan-cancer analysis of TOR1B for the first time. We discovered that TOR1B is aberrantly expressed in various malignant tumors, and the expression levels of TOR1B in these malignant tumor tissues are associated with patient prognosis. Therefore, investigating the role and mechanisms of TOR1B in the progression of malignant tumors, particularly in breast cancer, can offer new insights for developing novel targeted therapies for malignant tumors.

In this study, we focused on the role of TOR1B in breast cancer and further investigated the impact of TOR1B expression on the prognosis of the four PAM50 subtype breast cancers. Breast cancer molecular typing using PAM50 has several advantages over traditional typing methods. The PAM50 assay is a gene expression-based test that offers a more precise categorization of breast cancer subtypes in contrast to conventional approaches. Traditional typing methods rely on histological and immunohistochemical features of the tumor, which may not always accurately reflect the underlying molecular subtype (Kleivi Sahlberg et al. 2015). Through comprehensive analysis across multiple datasets, we found that the expression level of TOR1B in tumors significantly affects the prognosis of patients with BLBC, making it a risk factor for OS, DFS, and DRFS in these patients. We believe that TOR1B represents one of the potential therapeutic targets for BLBC.

Hypoxia, characterized by reduced oxygen availability, plays a significant role in breast cancer progression and resistance to treatment. In the tumor microenvironment, hypoxic conditions can lead to the stabilization and activation of hypoxia-inducible factors, which in turn activate a range of genes that promote angiogenesis, cell survival, proliferation, and metastasis (Wang et al. 2021). This adaptation allows breast cancer cells to survive under low oxygen conditions, contributes to an aggressive phenotype, and fosters resistance to conventional therapies such as radiation and chemotherapy (Jia et al. 2015). The HIF-1α is the primary transcription factor that is activated when hypoxia is present (Barsoum et al. 2014; Wahba and El-Hadaad 2015; Wan et al. 2020). HIF-1α significantly contributes to tumor development and progression, including breast cancer, through its pivotal roles in response to hypoxic conditions within tumors. It facilitates tumor growth by promoting angiogenesis, which ensures an increased blood supply to oxygen-deprived tumor areas, crucial for tumor survival and expansion. HIF-1α also drives metabolic reprogramming, shifting energy production from oxygen-reliant processes to glycolysis, enabling cancer cells to thrive in low-oxygen environments. Additionally, it plays a key role in metastasis by inducing epithelial-mesenchymal transition (EMT), enhancing the migratory and invasive capabilities of cancer cells. In breast cancer, HIF-1α's regulation of these processes is particularly critical, influencing tumor aggressiveness, therapeutic resistance, and overall prognosis. HIF-1α has been found to be excessively expressed in various types of tumors, such as ovarian, lung, gastric, breast, pancreatic, and prostate cancer, within a clinical environment. This overexpression has been associated with a poorer prognosis and the development of resistance to radiotherapy and chemotherapy, as indicated by studies (Shen et al. 2017; Wang et al. 2019; Jin et al. 2020; Wan et al. 2020; Sun et al. 2021).

In this study, through in vitro experiments, we further discovered that under hypoxic conditions, the expression levels of TOR1B and HIF1A in BLBC cells are higher than under normoxic conditions. Regardless of the oxygen levels, knocking out HIF1A resulted in a simultaneous decrease in the expression level of TOR1B, indicating that HIF1A may regulate the expression of TOR1B in BLBC cells. We believe that the change in TOR1B expression could be part of the metabolic reprogramming induced by HIF1A. This adaptation would make BLBC cells more tolerant to the hypoxic environment present within malignant lesions and more capable of adapting to the tumor microenvironment after metastasis, benefiting the progression of primary BLBC lesions and the occurrence of distant metastases. High TOR1B expression could lead to a poorer prognosis for BLBC patients, which also explains why survival analysis from public databases suggests that high TOR1B expression results in lower OS and DRFS for BLBC patients.

We further explored the impact of TOR1B on apoptosis in BLBC cells, finding that knocking out TOR1B induces apoptosis in BLBC under both hypoxic and normoxic conditions. Interestingly, under hypoxic conditions, the apoptosis rate in BLBC cells with TOR1B knocked out was higher than under normoxic conditions. Previous research has confirmed that knocking out HIF1A induces apoptosis in BLBC (Shi et al. 2010), and with TOR1B being a potential target gene of HIF1A, it may play a significant role in the anti-apoptotic process in BLBC under hypoxic conditions.

## Conclusion

Our research revealed that TOR1B plays a critical role in cancer progression, particularly in BLBC, where its aberrant expression correlates with poor prognosis. By analyzing TOR1B's interaction with metabolic pathways and its regulation by HIF-1α, we identified it as a key player in cancer cell adaptation to hypoxia. These findings highlight TOR1B's potential as a novel therapeutic target for BLBC, offering a new direction for targeted cancer therapy development.

## Supplementary Information

Below is the link to the electronic supplementary material.

Supplementary file1 (XLSX 666 KB)

Supplementary file2 (DOCX 22 KB)

Supplementary file3 (XLSX 56 KB)

Supplementary file4 (XLSX 13 KB)

Supplementary file5 (XLSX 50 KB)

Supplementary file6 (DOCX 18 KB)

Supplementary file7 (DOCX 12 KB)

Supplementary file8 (DOCX 49 KB)

Supplementary file9 (DOCX 31 KB)

Supplementary file10 (XLSX 194 KB)

## Data Availability

The datasets generated during and/or analysed during the current study are available from the corresponding author on reasonable request.

## Abbreviations

ACC: Adrenocortical cancer
BRCA: Breast invasive carcinoma
CESC: Cervical and endocervical cancer
CHOL: Cholangiocarcinoma
COAD: Colon adenocarcinoma
ESCA: Esophageal carcinoma
GBM: Glioblastoma multiforme
HNSC: Head and neck squamous cell carcinoma
KIRC: Kidney clear cell carcinoma
KIRP: Kidney papillary cell carcinoma
LAML: Acute myeloid leukemia
LGG: Brain lower grade glioma
LIHC: Liver hepatocellular carcinoma
LUAD: Lung adenocarcinoma
LUSC: Lung squamous cell carcinoma
OV: Ovarian serous cystadenocarcinoma
PAAD: Pancreatic adenocarcinoma
PRAD: Prostate adenocarcinoma
SKCM: Skin cutaneous melanoma
STAD: Stomach adenocarcinoma;
TGCT: Testicular germ cell tumor
THCA: Thyroid carcinoma
STES: Stomach and esophageal carcinoma
GBMLGG: Glioblastoma multiforme/lower grade glioma
KIPAN: Pankidney cohort
COADREAD: Colon adenocarcinoma/rectum adenocarcinoma esophageal carcinoma
TCGA: The cancer genome atlas
GEO: Gene expression omnibus
TARGET: Therapeutically applicable research to generate effective treatments
GTEx: The genotype-tissue expression project
BLBC: Basal-like breast cancer
ER: Endoplasmic reticulum
AAA +: ATPases associated with diverse cellular activities
ssGSEA: Single-sample gene set enrichment analysis
IHC: Immunohistochemistry
HPA: The human protein atlas
DFS: Disease-free survival
OS: Overall survival
DRFS: Disease and recurrence free survival
HR: Hazard ratios
CI: Confidence intervals
BM: Bone metastasis
BC: Breast cancer
PAM50: 50-Gene set predictor using predication analysis of microarray method
EMT: Epithelial-mesenchymal transition
