# Supplementary material for: Prognostic implications of TOR1B expression across cancer types: a focus on basal-like breast cancer and cellular adaptations to hypoxia
Source: J Cancer Res Clin Oncol. 2024 Jun 6;150(6):293. doi: 10.1007/s00432-024-05794-3 (PMC11156733; doi:10.1007/s00432-024-05794-3)
Supplement: Supplementary file 2 — Supplementary file2 (DOCX 22 KB) [file 432_2024_5794_MOESM2_ESM.docx]

| ID | HIF1A | TOR1B |
| --- | --- | --- |
| TCGA-A1-A0SK-01 | 7.082290642 | 5.491837066 |
| TCGA-A1-A0SO-01 | 7.297262045 | 5.024882568 |
| TCGA-A1-A0SP-01 | 6.274317579 | 5.307151618 |
| TCGA-A2-A04P-01 | 6.292700779 | 3.48955578 |
| TCGA-A2-A04Q-01 | 6.261164077 | 4.99809619 |
| TCGA-A2-A04T-01 | 5.941341105 | 4.887237678 |
| TCGA-A2-A04U-01 | 5.639614207 | 4.739416093 |
| TCGA-A2-A0CM-01 | 7.156966883 | 5.546054829 |
| TCGA-A2-A0D0-01 | 6.715345668 | 5.112512487 |
| TCGA-A2-A0D2-01 | 6.90551096 | 4.817039922 |
| TCGA-A2-A0ST-01 | 6.007487061 | 4.796665236 |
| TCGA-A2-A0SX-01 | 6.915133644 | 5.232419008 |
| TCGA-A2-A0T0-01 | 6.406927767 | 5.183411083 |
| TCGA-A2-A0T2-01 | 5.793596807 | 4.578220205 |
| TCGA-A2-A0YE-01 | 7.179822526 | 4.813216827 |
| TCGA-A2-A0YJ-01 | 6.53761135 | 5.677527974 |
| TCGA-A2-A0YM-01 | 7.686240679 | 6.166094776 |
| TCGA-A2-A1G1-01 | 7.375698054 | 5.483170853 |
| TCGA-A2-A25F-01 | 7.374999452 | 5.304408823 |
| TCGA-A7-A0CE-01 | 6.52922126 | 4.990709854 |
| TCGA-A7-A0DA-01 | 6.355752793 | 5.157266082 |
| TCGA-A7-A13D-01 | 6.851439235 | 4.431925996 |
| TCGA-A7-A13E-01 | 6.676642591 | 4.904408629 |
| TCGA-A7-A26F-01 | 7.651838858 | 4.505414609 |
| TCGA-A7-A26G-01 | 7.583756746 | 5.376467514 |
| TCGA-A7-A26I-01 | 6.150498751 | 4.599163041 |
| TCGA-A8-A07C-01 | 6.289332828 | 5.503736709 |
| TCGA-A8-A07O-01 | 6.665209409 | 5.745906884 |
| TCGA-A8-A07R-01 | 6.374203121 | 5.605177243 |
| TCGA-A8-A07U-01 | 6.205167661 | 4.801096559 |
| TCGA-A8-A08R-01 | 6.207863576 | 5.268943349 |
| TCGA-AC-A2BK-01 | 7.046164706 | 5.972040077 |
| TCGA-AC-A2QH-01 | 6.471075254 | 3.626871296 |
| TCGA-AN-A04D-01 | 5.851269312 | 4.220275775 |
| TCGA-AN-A0AL-01 | 7.529723358 | 5.445306253 |
| TCGA-AN-A0AT-01 | 5.814209448 | 4.661846491 |
| TCGA-AN-A0FJ-01 | 8.244584088 | 4.637581267 |
| TCGA-AN-A0FL-01 | 5.887010975 | 4.893405899 |
| TCGA-AN-A0FX-01 | 6.347711714 | 5.393762829 |
| TCGA-AN-A0G0-01 | 7.25377848 | 5.468677799 |
| TCGA-AN-A0XU-01 | 7.898577808 | 4.740031667 |
| TCGA-AO-A0J4-01 | 6.309734629 | 5.21600468 |
| TCGA-AO-A0J6-01 | 7.275810701 | 6.008446551 |
| TCGA-AO-A0JL-01 | 5.254813212 | 3.771294016 |
| TCGA-AO-A124-01 | 7.025548222 | 4.968680615 |
| TCGA-AO-A128-01 | 7.838098979 | 5.728668829 |
| TCGA-AO-A129-01 | 5.587973987 | 4.184089827 |
| TCGA-AO-A12F-01 | 7.428525162 | 4.614215121 |
| TCGA-AO-A1KR-01 | 4.000937447 | 3.732388605 |
| TCGA-AQ-A04J-01 | 6.907161076 | 4.183867583 |
| TCGA-AR-A0TP-01 | 5.855885078 | 4.329633115 |
| TCGA-AR-A0TS-01 | 6.857410023 | 5.635034605 |
| TCGA-AR-A0TU-01 | 6.214433755 | 4.698351849 |
| TCGA-AR-A0U0-01 | 7.368435744 | 5.298782933 |
| TCGA-AR-A0U4-01 | 8.516653042 | 5.137663359 |
| TCGA-AR-A1AH-01 | 5.880994012 | 4.520981617 |
| TCGA-AR-A1AI-01 | 6.912019865 | 4.796961081 |
| TCGA-AR-A1AJ-01 | 7.127550743 | 5.930179072 |
| TCGA-AR-A1AQ-01 | 5.658365743 | 4.281661162 |
| TCGA-AR-A1AR-01 | 7.702433836 | 5.88881873 |
| TCGA-AR-A1AY-01 | 7.598817763 | 5.201414304 |
| TCGA-AR-A24Q-01 | 6.247933208 | 5.838540878 |
| TCGA-AR-A251-01 | 7.573583571 | 5.257738573 |
| TCGA-AR-A256-01 | 7.104290526 | 5.088192491 |
| TCGA-AR-A2LR-01 | 6.810473952 | 5.040918725 |
| TCGA-B6-A0I1-01 | 5.156983057 | 5.075558302 |
| TCGA-B6-A0I2-01 | 5.099505617 | 4.624118446 |
| TCGA-B6-A0I6-01 | 6.63691458 | 5.154534604 |
| TCGA-B6-A0IJ-01 | 6.11424624 | 4.959946309 |
| TCGA-B6-A0IQ-01 | 5.4029336 | 4.582122407 |
| TCGA-B6-A0RE-01 | 7.039477308 | 4.881840786 |
| TCGA-B6-A0RT-01 | 6.38915721 | 5.009915932 |
| TCGA-B6-A0RU-01 | 7.191779765 | 5.507334807 |
| TCGA-B6-A0WX-01 | 6.039699918 | 5.240173106 |
| TCGA-B6-A0X1-01 | 6.437841939 | 5.59476649 |
| TCGA-B6-A1KF-01 | 7.934851425 | 5.951573808 |
| TCGA-BH-A0AV-01 | 6.909743108 | 5.430777017 |
| TCGA-BH-A0B3-01 | 6.161218568 | 4.767803085 |
| TCGA-BH-A0BG-01 | 5.909674713 | 4.624598088 |
| TCGA-BH-A0BL-01 | 7.312963708 | 5.052450674 |
| TCGA-BH-A0BW-01 | 7.382099158 | 6.024406121 |
| TCGA-BH-A0DL-01 | 7.423533632 | 5.119605182 |
| TCGA-BH-A0E0-01 | 5.746312766 | 3.260642961 |
| TCGA-BH-A0E6-01 | 4.739983079 | 4.282447219 |
| TCGA-BH-A0RX-01 | 6.50306723 | 5.098208987 |
| TCGA-BH-A0WA-01 | 7.203116957 | 5.621533235 |
| TCGA-BH-A18G-01 | 6.860311055 | 4.894934144 |
| TCGA-BH-A18Q-01 | 6.990082401 | 4.856861228 |
| TCGA-BH-A18T-01 | 7.039316087 | 5.007702329 |
| TCGA-BH-A18V-01 | 7.006186922 | 5.643423316 |
| TCGA-BH-A18V-06 | 5.764266527 | 5.39113874 |
| TCGA-BH-A1F0-01 | 7.319769652 | 5.788964932 |
| TCGA-BH-A1F6-01 | 6.380767538 | 5.633840943 |
| TCGA-BH-A1FC-01 | 6.166874085 | 5.529586724 |
| TCGA-C8-A12K-01 | 6.049861615 | 4.841857417 |
| TCGA-C8-A12V-01 | 6.32782746 | 5.191460003 |
| TCGA-C8-A131-01 | 7.353150355 | 5.225687532 |
| TCGA-C8-A134-01 | 6.614862938 | 4.897390497 |
| TCGA-C8-A1HJ-01 | 7.233981024 | 4.532765783 |
| TCGA-C8-A27B-01 | 6.742915695 | 5.531259801 |
| TCGA-D8-A142-01 | 6.395647233 | 5.076995121 |
| TCGA-D8-A143-01 | 7.980123579 | 5.064119752 |
| TCGA-D8-A147-01 | 6.733836966 | 4.952412782 |
| TCGA-D8-A1JK-01 | 7.741842443 | 4.847786498 |
| TCGA-D8-A1JL-01 | 8.931308094 | 5.592041362 |
| TCGA-D8-A1JM-01 | 6.299010149 | 6.02672925 |
| TCGA-D8-A1XK-01 | 7.127337158 | 5.710236129 |
| TCGA-D8-A1XQ-01 | 6.887203553 | 4.824783125 |
| TCGA-D8-A27F-01 | 7.146546275 | 4.664369059 |
| TCGA-D8-A27H-01 | 6.481035711 | 5.18657979 |
| TCGA-D8-A27M-01 | 6.571086689 | 5.457620556 |
| TCGA-E2-A14N-01 | 6.953241955 | 5.269455866 |
| TCGA-E2-A14R-01 | 5.600245962 | 4.098933223 |
| TCGA-E2-A14X-01 | 5.884363476 | 4.099926351 |
| TCGA-E2-A14Y-01 | 8.088064723 | 4.943166503 |
| TCGA-E2-A150-01 | 7.100671799 | 5.070904484 |
| TCGA-E2-A158-01 | 5.181078691 | 4.865117095 |
| TCGA-E2-A159-01 | 7.127675578 | 4.874978889 |
| TCGA-E2-A1AZ-01 | 7.320277971 | 4.894012539 |
| TCGA-E2-A1II-01 | 6.643354045 | 5.123078472 |
| TCGA-E2-A1LG-01 | 6.202848663 | 4.301258394 |
| TCGA-E2-A1LH-01 | 4.952580518 | 4.09927837 |
| TCGA-E2-A1LI-01 | 6.205785431 | 4.676075548 |
| TCGA-E2-A1LK-01 | 5.637303035 | 5.553461882 |
| TCGA-E2-A1LL-01 | 9.063177717 | 4.963746916 |
| TCGA-E2-A1LS-01 | 4.06547753 | 3.460572208 |
| TCGA-E9-A1N8-01 | 5.967168608 | 5.321509653 |
| TCGA-E9-A1N9-01 | 6.703563099 | 5.123434446 |
| TCGA-E9-A1ND-01 | 7.658437868 | 5.122780379 |
| TCGA-E9-A22G-01 | 5.27657855 | 5.027277928 |
| TCGA-E9-A243-01 | 7.044869475 | 4.926938763 |
| TCGA-E9-A244-01 | 6.00293875 | 5.025786276 |
| TCGA-EW-A1OW-01 | 7.018843336 | 5.14533508 |
| TCGA-EW-A1P4-01 | 7.333363799 | 4.870882517 |
| TCGA-EW-A1P8-01 | 6.512930045 | 6.091215329 |
| TCGA-EW-A1PB-01 | 5.710974451 | 5.49183386 |
| TCGA-EW-A1PH-01 | 6.326609251 | 5.273776954 |
| TCGA-GI-A2C9-01 | 6.43321987 | 5.157783468 |
| TCGA-GM-A2DF-01 | 5.519617384 | 3.820332161 |
| TCGA-HN-A2NL-01 | 5.5014901 | 4.546128934 |
