# Supplementary material for: Prognostic implications of TOR1B expression across cancer types: a focus on basal-like breast cancer and cellular adaptations to hypoxia
Source: J Cancer Res Clin Oncol. 2024 Jun 6;150(6):293. doi: 10.1007/s00432-024-05794-3 (PMC11156733; doi:10.1007/s00432-024-05794-3)
Supplement: Supplementary file 6 — Supplementary file6 (DOCX 18 KB) [file 432_2024_5794_MOESM6_ESM.docx]

| ID | Type | TOR1B |
| --- | --- | --- |
| GSM3943720 | Tumor(n=60) | 6.374386973 |
| GSM3943721 | Tumor(n=60) | 5.973690949 |
| GSM3943724 | Tumor(n=60) | 6.154431157 |
| GSM3943742 | Tumor(n=60) | 5.920874575 |
| GSM3943743 | Tumor(n=60) | 5.513045383 |
| GSM3943753 | Tumor(n=60) | 5.39514878 |
| GSM3943775 | Tumor(n=60) | 5.629994092 |
| GSM3943779 | Tumor(n=60) | 6.075789768 |
| GSM3943781 | Tumor(n=60) | 5.346009642 |
| GSM3943784 | Tumor(n=60) | 5.934861062 |
| GSM2301071 | Tumor(n=60) | 5.850084769 |
| GSM2301072 | Tumor(n=60) | 5.702672524 |
| GSM2301073 | Tumor(n=60) | 5.95732434 |
| GSM2301074 | Tumor(n=60) | 6.616084795 |
| GSM2301075 | Tumor(n=60) | 6.104710702 |
| GSM2301076 | Tumor(n=60) | 5.507779949 |
| GSM2301077 | Tumor(n=60) | 5.802063057 |
| GSM2301078 | Tumor(n=60) | 6.440398301 |
| GSM2301079 | Tumor(n=60) | 6.056480648 |
| GSM2301080 | Tumor(n=60) | 6.360525717 |
| GSM2301081 | Tumor(n=60) | 6.116934922 |
| GSM2301082 | Tumor(n=60) | 6.03749004 |
| GSM2301083 | Tumor(n=60) | 5.198380576 |
| GSM2301084 | Tumor(n=60) | 5.594562468 |
| GSM1403629 | Tumor(n=60) | 5.352757736 |
| GSM1403636 | Tumor(n=60) | 5.515808358 |
| GSM1403646 | Tumor(n=60) | 5.729280587 |
| GSM1403657 | Tumor(n=60) | 5.767090711 |
| GSM1403661 | Tumor(n=60) | 6.206888446 |
| GSM1403664 | Tumor(n=60) | 5.826551207 |
| GSM1403672 | Tumor(n=60) | 6.01025423 |
| GSM1403684 | Tumor(n=60) | 5.774743627 |
| GSM1403685 | Tumor(n=60) | 5.579450936 |
| GSM1403690 | Tumor(n=60) | 5.699055077 |
| GSM1403691 | Tumor(n=60) | 5.840879823 |
| GSM1403699 | Tumor(n=60) | 5.291558248 |
| GSM1403703 | Tumor(n=60) | 5.674870011 |
| GSM1403735 | Tumor(n=60) | 5.790508056 |
| GSM1403736 | Tumor(n=60) | 5.494791668 |
| GSM1403740 | Tumor(n=60) | 5.532298842 |
| GSM1403743 | Tumor(n=60) | 6.175084574 |
| GSM1403751 | Tumor(n=60) | 6.261209189 |
| GSM1403757 | Tumor(n=60) | 5.959690186 |
| GSM1403764 | Tumor(n=60) | 6.122023397 |
| GSM1403768 | Tumor(n=60) | 5.28050571 |
| GSM1403774 | Tumor(n=60) | 5.922337183 |
| GSM1403787 | Tumor(n=60) | 5.648642219 |
| GSM1403808 | Tumor(n=60) | 4.350522972 |
| GSM1403814 | Tumor(n=60) | 6.075447098 |
| GSM1403837 | Tumor(n=60) | 5.446690568 |
| GSM1403844 | Tumor(n=60) | 5.86251977 |
| GSM1403846 | Tumor(n=60) | 5.334002268 |
| GSM1403849 | Tumor(n=60) | 5.632167568 |
| GSM1403858 | Tumor(n=60) | 5.643403484 |
| GSM1403867 | Tumor(n=60) | 5.111182198 |
| GSM1403875 | Tumor(n=60) | 5.174232426 |
| GSM1403877 | Tumor(n=60) | 5.261418146 |
| GSM1403895 | Tumor(n=60) | 5.063616669 |
| GSM1403897 | Tumor(n=60) | 5.23352851 |
| GSM1403900 | Tumor(n=60) | 5.609858467 |
| GSM3943700 | Normal(n=37) | 5.322142454 |
| GSM3943701 | Normal(n=37) | 5.335691322 |
| GSM3943702 | Normal(n=37) | 5.130551996 |
| GSM3943703 | Normal(n=37) | 5.469023479 |
| GSM3943704 | Normal(n=37) | 5.386164045 |
| GSM3943705 | Normal(n=37) | 5.312565205 |
| GSM3943706 | Normal(n=37) | 5.346288962 |
| GSM3943707 | Normal(n=37) | 5.368096869 |
| GSM3943708 | Normal(n=37) | 5.390919302 |
| GSM3943709 | Normal(n=37) | 5.318777082 |
| GSM3943710 | Normal(n=37) | 5.350262451 |
| GSM3943711 | Normal(n=37) | 5.585473803 |
| GSM1403631 | Normal(n=37) | 5.316508276 |
| GSM1403653 | Normal(n=37) | 6.024648684 |
| GSM1403673 | Normal(n=37) | 5.510541692 |
| GSM1403687 | Normal(n=37) | 5.83116227 |
| GSM1403688 | Normal(n=37) | 5.730965698 |
| GSM1403709 | Normal(n=37) | 5.496789015 |
| GSM1403710 | Normal(n=37) | 6.234220512 |
| GSM1403724 | Normal(n=37) | 5.906667323 |
| GSM1403726 | Normal(n=37) | 5.674331772 |
| GSM1403733 | Normal(n=37) | 5.390398098 |
| GSM1403742 | Normal(n=37) | 5.736262782 |
| GSM1403753 | Normal(n=37) | 5.906087576 |
| GSM1403759 | Normal(n=37) | 5.307553515 |
| GSM1403770 | Normal(n=37) | 5.333182813 |
| GSM1403775 | Normal(n=37) | 6.106940817 |
| GSM1403785 | Normal(n=37) | 5.436381109 |
| GSM1403795 | Normal(n=37) | 4.818254192 |
| GSM1403816 | Normal(n=37) | 5.732087025 |
| GSM1403828 | Normal(n=37) | 5.396542973 |
| GSM1403832 | Normal(n=37) | 5.404581549 |
| GSM1403879 | Normal(n=37) | 5.576714552 |
| GSM1403890 | Normal(n=37) | 5.732939943 |
| GSM1403898 | Normal(n=37) | 5.469566795 |
| GSM1403899 | Normal(n=37) | 5.526262483 |
| GSM1403907 | Normal(n=37) | 5.129330785 |
