# Supplementary material for: Prognostic implications of TOR1B expression across cancer types: a focus on basal-like breast cancer and cellular adaptations to hypoxia
Source: J Cancer Res Clin Oncol. 2024 Jun 6;150(6):293. doi: 10.1007/s00432-024-05794-3 (PMC11156733; doi:10.1007/s00432-024-05794-3)
Supplement: Supplementary file 7 — Supplementary file7 (DOCX 12 KB) [file 432_2024_5794_MOESM7_ESM.docx]

| ID | Type | TOR1B |
| --- | --- | --- |
| GSM563551 | Turmor(n=16) | 7.244771646 |
| GSM563552 | Turmor(n=16) | 7.525602853 |
| GSM563553 | Turmor(n=16) | 7.350260063 |
| GSM563554 | Turmor(n=16) | 7.190253925 |
| GSM563557 | Turmor(n=16) | 7.191038972 |
| GSM563558 | Turmor(n=16) | 7.309169647 |
| GSM563559 | Turmor(n=16) | 7.339859731 |
| GSM563560 | Turmor(n=16) | 6.710583546 |
| GSM563561 | Turmor(n=16) | 7.114213693 |
| GSM563565 | Turmor(n=16) | 7.731699739 |
| GSM563566 | Turmor(n=16) | 7.374993066 |
| GSM563567 | Turmor(n=16) | 7.241481319 |
| GSM563568 | Turmor(n=16) | 7.752763161 |
| GSM563571 | Turmor(n=16) | 7.182717505 |
| GSM563572 | Turmor(n=16) | 6.927718745 |
| GSM563573 | Turmor(n=16) | 7.660118937 |
| GSM563555 | Normal(n=8) | 6.414511359 |
| GSM563556 | Normal(n=8) | 6.695167855 |
| GSM563562 | Normal(n=8) | 7.07786423 |
| GSM563563 | Normal(n=8) | 6.748923492 |
| GSM563564 | Normal(n=8) | 6.945181377 |
| GSM563569 | Normal(n=8) | 6.754631457 |
| GSM563570 | Normal(n=8) | 7.110419719 |
| GSM563574 | Normal(n=8) | 6.707041061 |
