# Supplementary material for: Prognostic implications of TOR1B expression across cancer types: a focus on basal-like breast cancer and cellular adaptations to hypoxia
Source: J Cancer Res Clin Oncol. 2024 Jun 6;150(6):293. doi: 10.1007/s00432-024-05794-3 (PMC11156733; doi:10.1007/s00432-024-05794-3)
Supplement: Supplementary file 8 — Supplementary file8 (DOCX 49 KB) [file 432_2024_5794_MOESM8_ESM.docx]

| ID | Type | DRFS time | DRFS event | TOR1B |
| --- | --- | --- | --- | --- |
| GSM615098 | Basal-like | 5.5 | 0 | 9.612898297 |
| GSM615099 | Basal-like | 5.42 | 0 | 9.513573493 |
| GSM615101 | Basal-like | 5.21 | 0 | 9.340575764 |
| GSM615102 | Basal-like | 2.73 | 1 | 9.49195509 |
| GSM615104 | Basal-like | 0.78 | 1 | 10.58277892 |
| GSM615106 | Basal-like | 4.16 | 0 | 9.554832917 |
| GSM615107 | Basal-like | 4.37 | 0 | 10.34936584 |
| GSM615109 | Basal-like | 1.25 | 1 | 9.652869666 |
| GSM615110 | Basal-like | 0.95 | 1 | 10.15664686 |
| GSM615112 | Basal-like | 1.83 | 1 | 9.104310914 |
| GSM615113 | Basal-like | 0.31 | 1 | 9.991018853 |
| GSM615116 | Basal-like | 0.96 | 1 | 10.19636056 |
| GSM615117 | Basal-like | 0.12 | 1 | 9.613250391 |
| GSM615118 | Basal-like | 2.42 | 0 | 9.487973125 |
| GSM615119 | Basal-like | 2.87 | 1 | 9.18159279 |
| GSM615124 | Basal-like | 4.16 | 0 | 10.45858748 |
| GSM615136 | Basal-like | 0.55 | 1 | 10.03528044 |
| GSM615137 | Basal-like | 2.46 | 0 | 10.01136821 |
| GSM615140 | Basal-like | 1.03 | 0 | 9.733796232 |
| GSM615143 | Basal-like | 2.9 | 0 | 10.00214322 |
| GSM615145 | Basal-like | 0.9 | 0 | 9.338259555 |
| GSM615146 | Basal-like | 2.53 | 0 | 9.784064983 |
| GSM615148 | Basal-like | 2.67 | 0 | 10.05173524 |
| GSM615149 | Basal-like | 0.72 | 1 | 10.19786869 |
| GSM615151 | Basal-like | 0.91 | 0 | 10.55147549 |
| GSM615152 | Basal-like | 2.8 | 0 | 9.839334573 |
| GSM615154 | Basal-like | 2.53 | 0 | 9.766654553 |
| GSM615155 | Basal-like | 3.19 | 0 | 9.709687498 |
| GSM615156 | Basal-like | 2.03 | 0 | 9.490015547 |
| GSM615160 | Basal-like | 0.38 | 1 | 9.175484357 |
| GSM615164 | Basal-like | 1.43 | 0 | 9.103840288 |
| GSM615169 | Basal-like | 2.26 | 0 | 9.272110163 |
| GSM615171 | Basal-like | 2.39 | 0 | 10.16573604 |
| GSM615175 | Basal-like | 2.53 | 0 | 9.733423796 |
| GSM615178 | Basal-like | 1.94 | 0 | 9.868901946 |
| GSM615181 | Basal-like | 7.4 | 0 | 10.27394082 |
| GSM615184 | Basal-like | 7.3 | 0 | 9.845785879 |
| GSM615185 | Basal-like | 5.59 | 1 | 10.28433416 |
| GSM615190 | Basal-like | 7.13 | 0 | 10.10725139 |
| GSM615191 | Basal-like | 6.34 | 0 | 9.685474393 |
| GSM615194 | Basal-like | 6.52 | 0 | 9.256815372 |
| GSM615195 | Basal-like | 0.76 | 1 | 9.757710824 |
| GSM615202 | Basal-like | 6.45 | 0 | 9.293894146 |
| GSM615209 | Basal-like | 2.13 | 1 | 10.3257907 |
| GSM615211 | Basal-like | 5.14 | 0 | 9.418547014 |
| GSM615212 | Basal-like | 4.68 | 0 | 9.534882208 |
| GSM615213 | Basal-like | 4.62 | 0 | 9.623474137 |
| GSM615218 | Basal-like | 0.58 | 1 | 9.962398191 |
| GSM615219 | Basal-like | 0.48 | 0 | 10.30454366 |
| GSM615223 | Basal-like | 0.62 | 1 | 9.298492711 |
| GSM615225 | Basal-like | 4.33 | 0 | 9.5006838 |
| GSM615240 | Basal-like | 3.8 | 0 | 10.20523322 |
| GSM615242 | Basal-like | 4.49 | 0 | 9.909290104 |
| GSM615244 | Basal-like | 4.06 | 0 | 9.740142178 |
| GSM615248 | Basal-like | 3.21 | 0 | 9.699016611 |
| GSM615251 | Basal-like | 3.75 | 0 | 9.466366265 |
| GSM615255 | Basal-like | 0.85 | 1 | 10.13819575 |
| GSM615260 | Basal-like | 1.75 | 1 | 9.324716439 |
| GSM615264 | Basal-like | 3.73 | 0 | 9.746441498 |
| GSM615270 | Basal-like | 3.72 | 0 | 9.529631954 |
| GSM615275 | Basal-like | 2.4 | 1 | 10.23714283 |
| GSM615276 | Basal-like | 3.27 | 0 | 9.932139516 |
| GSM615284 | Basal-like | 2.84 | 0 | 9.850353129 |
| GSM615288 | Basal-like | 2.6 | 0 | 9.563608237 |
| GSM615290 | Basal-like | 3.15 | 0 | 9.576921481 |
| GSM615291 | Basal-like | 0.71 | 1 | 9.500965674 |
| GSM615293 | Basal-like | 1.37 | 1 | 9.582402647 |
| GSM615297 | Basal-like | 2.34 | 1 | 9.784434815 |
| GSM615300 | Basal-like | 1.88 | 0 | 10.14041452 |
| GSM615301 | Basal-like | 2.55 | 1 | 9.814786932 |
| GSM615303 | Basal-like | 2.34 | 0 | 9.233290343 |
| GSM615307 | Basal-like | 2.82 | 0 | 9.788289043 |
| GSM615310 | Basal-like | 2.34 | 0 | 9.3443553 |
| GSM615312 | Basal-like | 1.71 | 1 | 9.856798096 |
| GSM615315 | Basal-like | 0.89 | 1 | 9.206145122 |
| GSM615317 | Basal-like | 2.7 | 0 | 9.142297366 |
| GSM615319 | Basal-like | 2.28 | 1 | 9.55043014 |
| GSM615320 | Basal-like | 1.35 | 1 | 9.727577339 |
| GSM615321 | Basal-like | 2.52 | 0 | 8.964274368 |
| GSM615322 | Basal-like | 0.8 | 1 | 10.17985056 |
| GSM615324 | Basal-like | 2.46 | 0 | 9.884152584 |
| GSM615325 | Basal-like | 2.15 | 0 | 10.22350809 |
| GSM615326 | Basal-like | 2.48 | 0 | 9.92931505 |
| GSM615327 | Basal-like | 2.53 | 1 | 9.869979546 |
| GSM615330 | Basal-like | 0.8 | 1 | 10.34882416 |
| GSM615331 | Basal-like | 2.19 | 0 | 9.868169964 |
| GSM615333 | Basal-like | 2.23 | 0 | 9.758168662 |
| GSM615334 | Basal-like | 2.2 | 0 | 9.806822028 |
| GSM615335 | Basal-like | 0.92 | 1 | 9.672088293 |
| GSM615336 | Basal-like | 2.26 | 0 | 9.571462424 |
| GSM615338 | Basal-like | 2.06 | 0 | 10.00369799 |
| GSM615346 | Basal-like | 1.31 | 0 | 9.466794319 |
| GSM615347 | Basal-like | 2.25 | 1 | 9.463327141 |
| GSM615348 | Basal-like | 2.3 | 0 | 10.17177874 |
| GSM615354 | Basal-like | 1.42 | 0 | 9.596214338 |
| GSM615357 | Basal-like | 1.74 | 0 | 9.231386916 |
| GSM615359 | Basal-like | 1.73 | 0 | 9.098551907 |
| GSM615363 | Basal-like | 1.61 | 0 | 9.806956887 |
| GSM615364 | Basal-like | 1.54 | 0 | 10.13206964 |
| GSM615368 | Basal-like | 0.73 | 1 | 9.963974634 |
| GSM615369 | Basal-like | 1.9 | 0 | 9.576251285 |
| GSM615371 | Basal-like | 1.71 | 0 | 9.267532376 |
| GSM615372 | Basal-like | 1.7 | 0 | 9.482484231 |
| GSM615375 | Basal-like | 1.24 | 0 | 9.783135328 |
| GSM615376 | Basal-like | 1.13 | 1 | 9.527227052 |
| GSM615378 | Basal-like | 1.12 | 0 | 10.11650608 |
| GSM615379 | Basal-like | 1.14 | 0 | 9.640411784 |
| GSM615380 | Basal-like | 0.17 | 1 | 9.970640348 |
| GSM615382 | Basal-like | 1.14 | 0 | 9.37472076 |
| GSM615384 | Basal-like | 1.28 | 0 | 8.911526995 |
| GSM615385 | Basal-like | 1.13 | 0 | 10.1394877 |
| GSM615387 | Basal-like | 1.39 | 0 | 9.718125554 |
| GSM615390 | Basal-like | 1.49 | 0 | 9.3899344 |
| GSM615392 | Basal-like | 0.09 | 1 | 9.451867811 |
| GSM615393 | Basal-like | 1.27 | 0 | 9.340047722 |
| GSM615394 | Basal-like | 1.19 | 0 | 9.323798678 |
| GSM615396 | Basal-like | 0.82 | 0 | 9.724106144 |
| GSM615397 | Basal-like | 0.93 | 0 | 9.620199381 |
| GSM615403 | Basal-like | 1.27 | 1 | 9.251575982 |
| GSM615632 | Basal-like | 5.14 | 0 | 10.0578679 |
| GSM615637 | Basal-like | 4.94 | 0 | 10.39633744 |
| GSM615638 | Basal-like | 3.49 | 1 | 9.298086244 |
| GSM615639 | Basal-like | 1.44 | 1 | 9.884567051 |
| GSM615640 | Basal-like | 1.19 | 1 | 8.965785114 |
| GSM615641 | Basal-like | 4.46 | 0 | 9.744798809 |
| GSM615643 | Basal-like | 4.82 | 0 | 9.88788242 |
| GSM615644 | Basal-like | 4.46 | 0 | 9.559388237 |
| GSM615648 | Basal-like | 4.42 | 0 | 9.036998107 |
| GSM615649 | Basal-like | 0.45 | 1 | 10.39518231 |
| GSM615650 | Basal-like | 4.77 | 0 | 9.965266305 |
| GSM615651 | Basal-like | 0.79 | 1 | 10.30936609 |
| GSM615657 | Basal-like | 0.42 | 1 | 10.1450294 |
| GSM615658 | Basal-like | 4.48 | 0 | 9.144077342 |
| GSM615660 | Basal-like | 3.05 | 0 | 9.616605223 |
| GSM615661 | Basal-like | 4.43 | 0 | 9.455937041 |
| GSM615666 | Basal-like | 4.57 | 0 | 9.277954832 |
| GSM615667 | Basal-like | 2.22 | 0 | 10.27788857 |
| GSM615668 | Basal-like | 1.51 | 1 | 9.963013077 |
| GSM615671 | Basal-like | 3.96 | 0 | 9.533346681 |
| GSM615672 | Basal-like | 2.86 | 0 | 9.558906233 |
| GSM615674 | Basal-like | 0.47 | 1 | 8.778709104 |
| GSM615676 | Basal-like | 4.19 | 0 | 9.515635789 |
| GSM615677 | Basal-like | 4.06 | 0 | 9.43858191 |
| GSM615680 | Basal-like | 3.93 | 0 | 9.883584925 |
| GSM615681 | Basal-like | 1.23 | 1 | 9.443526955 |
| GSM615687 | Basal-like | 1.26 | 1 | 9.751569479 |
| GSM615689 | Basal-like | 2.03 | 1 | 10.18435166 |
| GSM615691 | Basal-like | 2.1 | 1 | 10.45314877 |
| GSM615694 | Basal-like | 5.41 | 0 | 9.82775278 |
| GSM615695 | Basal-like | 5.38 | 0 | 9.660259297 |
| GSM615696 | Basal-like | 2.95 | 1 | 10.35859788 |
| GSM615699 | Basal-like | 5.86 | 0 | 9.747209457 |
| GSM615701 | Basal-like | 5.3 | 0 | 9.769273327 |
| GSM615706 | Basal-like | 4.66 | 0 | 8.297700106 |
| GSM615707 | Basal-like | 4.79 | 0 | 9.595237233 |
| GSM615712 | Basal-like | 1.24 | 1 | 9.657932058 |
| GSM615714 | Basal-like | 2.85 | 0 | 10.39668085 |
| GSM615715 | Basal-like | 2.82 | 0 | 9.330024845 |
| GSM615716 | Basal-like | 2.49 | 0 | 10.09908479 |
| GSM615727 | Basal-like | 2.78 | 0 | 9.32074275 |
| GSM615728 | Basal-like | 2.82 | 0 | 9.041378055 |
| GSM615730 | Basal-like | 1.27 | 1 | 9.777056019 |
| GSM615732 | Basal-like | 1.63 | 1 | 10.33056863 |
| GSM615733 | Basal-like | 2.19 | 0 | 9.824456207 |
| GSM615737 | Basal-like | 2.57 | 0 | 10.26530748 |
| GSM615739 | Basal-like | 1.65 | 1 | 10.91226188 |
| GSM615741 | Basal-like | 1.63 | 0 | 9.433789544 |
| GSM615742 | Basal-like | 2.09 | 0 | 9.954246631 |
| GSM615748 | Basal-like | 1.82 | 0 | 10.33420598 |
| GSM615755 | Basal-like | 0.98 | 0 | 10.02788852 |
| GSM615757 | Basal-like | 0.76 | 1 | 10.19553232 |
| GSM615763 | Basal-like | 6.09 | 0 | 10.30270857 |
| GSM615764 | Basal-like | 5.59 | 0 | 9.506065077 |
| GSM615769 | Basal-like | 1.7 | 1 | 9.429405319 |
| GSM615773 | Basal-like | 4.94 | 0 | 9.379623134 |
| GSM615776 | Basal-like | 1.05 | 1 | 9.853445097 |
| GSM615786 | Basal-like | 1.6 | 0 | 9.997028544 |
| GSM615787 | Basal-like | 0.72 | 1 | 10.40289092 |
| GSM615794 | Basal-like | 3.35 | 1 | 10.18934249 |
| GSM615798 | Basal-like | 3.41 | 0 | 10.09839971 |
| GSM615800 | Basal-like | 0.88 | 1 | 10.19291075 |
| GSM615801 | Basal-like | 2.67 | 1 | 10.23146633 |
| GSM615804 | Basal-like | 3.13 | 0 | 9.865505414 |
| GSM615806 | Basal-like | 0.53 | 1 | 10.60445339 |
| GSM615813 | Basal-like | 3.7 | 0 | 9.531472077 |
| GSM615815 | Basal-like | 3.81 | 0 | 10.18361785 |

| GSM615096 | Luminal A | 2.35 | 1 | 9.37616342 |
| --- | --- | --- | --- | --- |
| GSM615100 | Luminal A | 1.58 | 1 | 10.56424192 |
| GSM615103 | Luminal A | 5.3 | 0 | 9.706207777 |
| GSM615105 | Luminal A | 0 | 1 | 10.26683798 |
| GSM615114 | Luminal A | 4.1 | 0 | 9.701608438 |
| GSM615120 | Luminal A | 4.71 | 0 | 10.14844382 |
| GSM615126 | Luminal A | 3.8 | 0 | 10.05393878 |
| GSM615127 | Luminal A | 4.13 | 0 | 9.850300791 |
| GSM615131 | Luminal A | 2.88 | 0 | 9.486236681 |
| GSM615132 | Luminal A | 1.57 | 0 | 9.647280142 |
| GSM615133 | Luminal A | 3.62 | 0 | 10.32388564 |
| GSM615142 | Luminal A | 2.95 | 0 | 10.51491611 |
| GSM615144 | Luminal A | 3.26 | 0 | 10.52198665 |
| GSM615147 | Luminal A | 2.87 | 0 | 11.15450709 |
| GSM615150 | Luminal A | 2.17 | 0 | 9.451660447 |
| GSM615158 | Luminal A | 2.28 | 0 | 9.26578897 |
| GSM615161 | Luminal A | 2.4 | 0 | 9.950228703 |
| GSM615162 | Luminal A | 2.51 | 0 | 9.90683169 |
| GSM615168 | Luminal A | 1.36 | 0 | 10.41097472 |
| GSM615170 | Luminal A | 2.2 | 0 | 9.974587151 |
| GSM615172 | Luminal A | 2.59 | 0 | 9.755708865 |
| GSM615173 | Luminal A | 2.09 | 0 | 9.910310995 |
| GSM615179 | Luminal A | 7.08 | 0 | 9.119407533 |
| GSM615180 | Luminal A | 5.76 | 0 | 9.742654368 |
| GSM615183 | Luminal A | 6.71 | 0 | 9.685745156 |
| GSM615186 | Luminal A | 6.66 | 0 | 10.22391613 |
| GSM615187 | Luminal A | 6.83 | 0 | 9.741050157 |
| GSM615192 | Luminal A | 7.33 | 0 | 9.396334731 |
| GSM615198 | Luminal A | 6.59 | 0 | 9.363089106 |
| GSM615199 | Luminal A | 6.54 | 0 | 9.583943365 |
| GSM615203 | Luminal A | 6.51 | 0 | 9.949538638 |
| GSM615204 | Luminal A | 6.31 | 0 | 9.447441922 |
| GSM615207 | Luminal A | 5.79 | 0 | 9.83140008 |
| GSM615208 | Luminal A | 5.87 | 0 | 9.36569259 |
| GSM615210 | Luminal A | 5.89 | 0 | 9.253259658 |
| GSM615214 | Luminal A | 5.54 | 0 | 10.0775682 |
| GSM615217 | Luminal A | 5.65 | 0 | 9.684247755 |
| GSM615221 | Luminal A | 3.36 | 1 | 9.499576052 |
| GSM615222 | Luminal A | 1.54 | 1 | 9.921353133 |
| GSM615224 | Luminal A | 4.6 | 0 | 9.767518962 |
| GSM615228 | Luminal A | 4.58 | 0 | 9.40603202 |
| GSM615231 | Luminal A | 4.53 | 0 | 9.883816572 |
| GSM615232 | Luminal A | 3.89 | 0 | 9.581659732 |
| GSM615236 | Luminal A | 2.88 | 1 | 10.36135115 |
| GSM615237 | Luminal A | 4.58 | 0 | 10.18066255 |
| GSM615239 | Luminal A | 4.11 | 0 | 9.886576976 |
| GSM615241 | Luminal A | 0.05 | 1 | 9.391829025 |
| GSM615245 | Luminal A | 2.26 | 1 | 9.805859078 |
| GSM615246 | Luminal A | 4.54 | 0 | 10.28861328 |
| GSM615247 | Luminal A | 4.03 | 0 | 9.422537469 |
| GSM615252 | Luminal A | 4.19 | 0 | 9.998078913 |
| GSM615254 | Luminal A | 4.15 | 0 | 9.36569259 |
| GSM615259 | Luminal A | 3.78 | 0 | 9.487489123 |
| GSM615261 | Luminal A | 2.97 | 0 | 10.00253644 |
| GSM615262 | Luminal A | 4.78 | 0 | 9.846969728 |
| GSM615266 | Luminal A | 3.52 | 0 | 9.672769232 |
| GSM615268 | Luminal A | 3.5 | 0 | 9.728960178 |
| GSM615273 | Luminal A | 2.28 | 0 | 9.20322782 |
| GSM615277 | Luminal A | 3.59 | 0 | 9.872465448 |
| GSM615278 | Luminal A | 3.25 | 0 | 10.39352587 |
| GSM615283 | Luminal A | 3.19 | 0 | 8.947164737 |
| GSM615285 | Luminal A | 3.37 | 0 | 10.01788313 |
| GSM615292 | Luminal A | 2.37 | 0 | 9.345661842 |
| GSM615296 | Luminal A | 2.11 | 0 | 9.35192044 |
| GSM615298 | Luminal A | 2.99 | 0 | 9.728960178 |
| GSM615299 | Luminal A | 2.67 | 0 | 9.713141796 |
| GSM615305 | Luminal A | 2.65 | 1 | 9.668954809 |
| GSM615306 | Luminal A | 1.82 | 1 | 10.42818814 |
| GSM615311 | Luminal A | 2.69 | 0 | 10.46243977 |
| GSM615314 | Luminal A | 2.26 | 0 | 11.52307671 |
| GSM615316 | Luminal A | 1.88 | 0 | 10.02528034 |
| GSM615318 | Luminal A | 2.37 | 0 | 8.918730675 |
| GSM615328 | Luminal A | 2.36 | 0 | 10.21950051 |
| GSM615329 | Luminal A | 2.06 | 0 | 9.199300172 |
| GSM615337 | Luminal A | 2.18 | 0 | 9.638610959 |
| GSM615339 | Luminal A | 2.17 | 0 | 9.330710292 |
| GSM615341 | Luminal A | 2.22 | 0 | 9.878831991 |
| GSM615349 | Luminal A | 2.33 | 0 | 9.681282668 |
| GSM615351 | Luminal A | 2.39 | 0 | 9.538329986 |
| GSM615352 | Luminal A | 2.16 | 0 | 9.934104699 |
| GSM615355 | Luminal A | 2.01 | 0 | 9.503855922 |
| GSM615360 | Luminal A | 1.98 | 0 | 9.594847219 |
| GSM615362 | Luminal A | 1.96 | 0 | 9.600530332 |
| GSM615365 | Luminal A | 1.93 | 0 | 9.856466192 |
| GSM615366 | Luminal A | 1.88 | 0 | 9.789013054 |
| GSM615370 | Luminal A | 1.34 | 0 | 10.61603435 |
| GSM615374 | Luminal A | 1.67 | 0 | 9.689676151 |
| GSM615381 | Luminal A | 1.01 | 0 | 9.715512709 |
| GSM615383 | Luminal A | 1.24 | 0 | 9.609526126 |
| GSM615386 | Luminal A | 0.88 | 0 | 9.541597354 |
| GSM615388 | Luminal A | 0.78 | 0 | 9.788600317 |
| GSM615389 | Luminal A | 1.15 | 0 | 9.690163419 |
| GSM615395 | Luminal A | 1.06 | 0 | 9.618653374 |
| GSM615399 | Luminal A | 1.05 | 0 | 10.1897778 |
| GSM615400 | Luminal A | 2.75 | 0 | 9.502961275 |
| GSM615402 | Luminal A | 2.36 | 0 | 9.568240261 |
| GSM615631 | Luminal A | 4.55 | 1 | 9.661023852 |
| GSM615642 | Luminal A | 4.83 | 0 | 9.453435068 |
| GSM615646 | Luminal A | 4.69 | 0 | 10.10468377 |
| GSM615647 | Luminal A | 4.52 | 0 | 9.909133632 |
| GSM615654 | Luminal A | 3.43 | 1 | 10.12869157 |
| GSM615659 | Luminal A | 4.42 | 0 | 9.588989217 |
| GSM615665 | Luminal A | 4.48 | 0 | 10.1378427 |
| GSM615675 | Luminal A | 4.1 | 0 | 9.710218206 |
| GSM615679 | Luminal A | 1.92 | 0 | 9.976889191 |
| GSM615682 | Luminal A | 3.7 | 0 | 9.571371634 |
| GSM615683 | Luminal A | 3.99 | 0 | 9.229680961 |
| GSM615685 | Luminal A | 7.36 | 0 | 10.23640152 |
| GSM615686 | Luminal A | 1.34 | 1 | 9.17682095 |
| GSM615688 | Luminal A | 6.61 | 0 | 10.28429934 |
| GSM615690 | Luminal A | 6.41 | 0 | 9.145746392 |
| GSM615692 | Luminal A | 4.14 | 0 | 9.5586455 |
| GSM615693 | Luminal A | 4.73 | 0 | 9.660122223 |
| GSM615697 | Luminal A | 6.08 | 0 | 9.183549142 |
| GSM615698 | Luminal A | 5.76 | 0 | 9.912095489 |
| GSM615700 | Luminal A | 6.02 | 0 | 9.370310614 |
| GSM615705 | Luminal A | 5.29 | 0 | 10.19604442 |
| GSM615710 | Luminal A | 4.06 | 0 | 8.696017075 |
| GSM615711 | Luminal A | 4.15 | 0 | 9.705136116 |
| GSM615713 | Luminal A | 3.09 | 1 | 9.455654886 |
| GSM615718 | Luminal A | 1.84 | 0 | 10.31414195 |
| GSM615719 | Luminal A | 3.1 | 0 | 9.429218327 |
| GSM615721 | Luminal A | 2.93 | 0 | 10.08144399 |
| GSM615722 | Luminal A | 3.02 | 0 | 10.24344551 |
| GSM615724 | Luminal A | 2.85 | 0 | 9.722028575 |
| GSM615725 | Luminal A | 3.03 | 0 | 10.63544318 |
| GSM615729 | Luminal A | 1.94 | 1 | 10.21525548 |
| GSM615734 | Luminal A | 2.81 | 0 | 9.328125252 |
| GSM615738 | Luminal A | 2.38 | 0 | 9.063937901 |
| GSM615740 | Luminal A | 2.32 | 0 | 9.642889595 |
| GSM615743 | Luminal A | 2.11 | 0 | 10.16172722 |
| GSM615745 | Luminal A | 1.95 | 0 | 9.433550077 |
| GSM615747 | Luminal A | 2.05 | 0 | 10.95615114 |
| GSM615750 | Luminal A | 1.69 | 0 | 10.12271648 |
| GSM615751 | Luminal A | 1.74 | 0 | 9.963631472 |
| GSM615752 | Luminal A | 1.35 | 0 | 9.569620605 |
| GSM615756 | Luminal A | 3.4 | 0 | 9.338186756 |
| GSM615758 | Luminal A | 4.17 | 1 | 9.199736272 |
| GSM615759 | Luminal A | 3.94 | 0 | 9.615273949 |
| GSM615761 | Luminal A | 5.02 | 0 | 9.312925458 |
| GSM615768 | Luminal A | 5.18 | 0 | 9.227395136 |
| GSM615770 | Luminal A | 1.69 | 0 | 9.632342595 |
| GSM615772 | Luminal A | 4.82 | 0 | 8.717362159 |
| GSM615777 | Luminal A | 4.02 | 0 | 10.08202568 |
| GSM615791 | Luminal A | 4.12 | 0 | 9.735844283 |
| GSM615797 | Luminal A | 3.48 | 0 | 9.916168051 |
| GSM615799 | Luminal A | 3.48 | 0 | 9.533268501 |
| GSM615807 | Luminal A | 3.91 | 0 | 9.829271852 |
| GSM615810 | Luminal A | 2.14 | 1 | 9.722028575 |
| GSM615811 | Luminal A | 3.42 | 0 | 10.02827365 |
| GSM615816 | Luminal A | 3.38 | 0 | 9.96068127 |
| GSM615818 | Luminal A | 2.93 | 0 | 10.52901236 |
| GSM615819 | Luminal A | 3.01 | 0 | 9.943006196 |
| GSM615820 | Luminal A | 1.21 | 1 | 10.1067196 |
| GSM615825 | Luminal A | 2.45 | 0 | 10.03381667 |
| GSM615826 | Luminal A | 3.18 | 0 | 9.648777269 |
| GSM615828 | Luminal A | 3.08 | 0 | 9.870353737 |
