# Supplementary material for: Prognostic implications of TOR1B expression across cancer types: a focus on basal-like breast cancer and cellular adaptations to hypoxia
Source: J Cancer Res Clin Oncol. 2024 Jun 6;150(6):293. doi: 10.1007/s00432-024-05794-3 (PMC11156733; doi:10.1007/s00432-024-05794-3)
Supplement: Supplementary file 9 — Supplementary file9 (DOCX 31 KB) [file 432_2024_5794_MOESM9_ESM.docx]

| ID | TOR1B | t | n | m | OS | OS.time | DFSstate | DFS_time |
| --- | --- | --- | --- | --- | --- | --- | --- | --- |
| TCGA-A1-A0SP | 5.30715161775304 | T2 | N0 | M0 | 0 | 584 | 0 | 584 |
| TCGA-A2-A04Q | 4.99809619031422 | T1 | N0 | M0 | 0 | 2385 | 0 | 2385 |
| TCGA-A2-A04T | 4.88723767786428 | T2 | N0 | M0 | 0 | 2246 | 0 | 2246 |
| TCGA-A2-A04U | 4.73941609340103 | T2 | N0 | M0 | 0 | 2654 | 0 | 2654 |
| TCGA-A2-A0CM | 5.54605482940219 | T2 | N0 | M0 | 1 | 754 | 0 | 754 |
| TCGA-A2-A0D0 | 5.11251248679859 | T2 | N0 | M0 | 0 | 2048 | 0 | 2048 |
| TCGA-A2-A0D2 | 4.81703992245115 | T2 | N0 | M0 | 0 | 1027 | 0 | 1027 |
| TCGA-A2-A0SX | 5.23241900837840 | T1 | N0 | M0 | 0 | 1534 | 1 | 1239 |
| TCGA-A2-A0T0 | 5.18341108264215 | T2 | N1 | M0 | 0 | 533 | 0 | 533 |
| TCGA-A2-A0T2 | 4.57822020487308 | T3 | N3 | M1 | 1 | 255 | 0 | 255 |
| TCGA-A2-A0YE | 4.81321682678480 | T2 | N1 | M0 | 0 | 554 | 0 | 554 |
| TCGA-A2-A0YJ | 5.67752797418388 | T3 | N2 | M0 | 0 | 566 | 1 | 559 |
| TCGA-A2-A1G1 | 5.48317085322987 | T2 | N1 | M0 | 0 | 584 | 0 | 584 |
| TCGA-A2-A25F | 5.30440882295311 | T2 | N0 | M0 | 0 | 322 | 0 | 322 |
| TCGA-A7-A0CE | 4.99070985364360 | T2 | N0 | M0 | 0 | 1074 | 0 | 1074 |
| TCGA-A7-A0DA | 5.15726608153625 | T2 | N0 | M0 | 0 | 1085 | 0 | 1085 |
| TCGA-A7-A13D | 4.43192599635183 | T2 | N0 | M0 | 0 | 965 | 0 | 965 |
| TCGA-A7-A26F | 4.50541460934675 | T1 | N0 | M0 | 0 | 738 | 0 | 738 |
| TCGA-A7-A26G | 5.37646751385118 | T2 | N0 | M0 | 0 | 722 | 0 | 722 |
| TCGA-A7-A26I | 4.59916304088104 | T2 | N0 | M0 | 0 | 661 | 0 | 661 |
| TCGA-A8-A07C | 5.50373670943915 | T2 | N0 | M0 | 0 | 1034 | 0 | 1034 |
| TCGA-A8-A07R | 5.60517724271224 | T2 | N3 | M0 | 0 | 273 | 0 | 273 |
| TCGA-A8-A07U | 4.80109655884450 | T2 | N2 | M0 | 0 | 760 | 0 | 760 |
| TCGA-A8-A08R | 5.26894334936354 | T2 | N1 | M0 | 0 | 30 | 0 | 30 |
| TCGA-AN-A04D | 4.22027577540039 | T2 | N1 | M0 | 0 | 52 | 0 | 52 |
| TCGA-AN-A0AL | 5.44530625325346 | T4 | N0 | M0 | 0 | 227 | 0 | 227 |
| TCGA-AN-A0FJ | 4.63758126684375 | T2 | N2 | M1 | 0 | 242 | 0 | 242 |
| TCGA-AN-A0FL | 4.89340589871398 | T2 | N0 | M0 | 0 | 231 | 0 | 231 |
| TCGA-AO-A0J4 | 5.21600468026895 | T1 | N0 | M0 | 0 | 1587 | 0 | 1587 |
| TCGA-AO-A0J6 | 6.00844655059658 | T2 | N0 | M0 | 0 | 1140 | 0 | 1140 |
| TCGA-AO-A0JL | 3.77129401565166 | T2 | N2 | M0 | 0 | 1683 | 0 | 1683 |
| TCGA-AO-A124 | 4.96868061535693 | T2 | N0 | M0 | 0 | 3506 | 0 | 3506 |
| TCGA-AO-A129 | 4.18408982705613 | T2 | N1 | M0 | 0 | 3286 | 0 | 3286 |
| TCGA-AO-A12F | 4.61421512100095 | T2 | N0 | M0 | 0 | 1842 | 0 | 1842 |
| TCGA-AO-A1KR | 3.73238860498474 | T2 | N0 | M0 | 0 | 2513 | 0 | 2513 |
| TCGA-AQ-A04J | 4.18386758334608 | T2 | N0 | M0 | 0 | 819 | 0 | 819 |
| TCGA-AR-A0TP | 4.32963311500328 | T2 | N0 | M0 | 0 | 4275 | 0 | 4275 |
| TCGA-AR-A0TS | 5.63503460521373 | T2 | N1 | M0 | 0 | 2558 | 0 | 2558 |
| TCGA-AR-A0TU | 4.69835184910483 | T2 | N0 | M0 | 0 | 709 | 0 | 709 |
| TCGA-AR-A0U0 | 5.29878293322946 | T2 | N1 | M0 | 0 | 1988 | 0 | 1988 |
| TCGA-AR-A0U4 | 5.13766335894842 | T2 | N0 | M0 | 0 | 3261 | 0 | 3261 |
| TCGA-AR-A1AH | 4.52098161658034 | T2 | N1 | M0 | 0 | 3807 | 1 | 1136 |
| TCGA-AR-A1AI | 4.79696108139768 | T2 | N0 | M0 | 0 | 3296 | 1 | 588 |
| TCGA-AR-A1AQ | 4.28166116234601 | T2 | N0 | M0 | 0 | 3021 | 0 | 3021 |
| TCGA-AR-A1AR | 5.88881872970218 | T1 | N2 | M0 | 1 | 524 | 1 | 268 |
| TCGA-AR-A1AY | 5.20141430391408 | T1 | N0 | M0 | 0 | 1026 | 0 | 1026 |
| TCGA-AR-A251 | 5.25773857296848 | T2 | N2 | M0 | 0 | 3030 | 0 | 3030 |
| TCGA-AR-A256 | 5.08819249066938 | T2 | N0 | M0 | 1 | 2854 | 1 | 1640 |
| TCGA-AR-A2LR | 5.04091872497060 | T1 | N0 | M0 | 0 | 1742 | 0 | 1742 |
| TCGA-B6-A0I1 | 5.07555830166924 | T2 | N0 | M0 | 1 | 2361 | 1 | 1120 |
| TCGA-B6-A0I2 | 4.62411844555592 | T1 | N0 | M0 | 0 | 4361 | 0 | 4361 |
| TCGA-B6-A0I6 | 5.15453460427778 | T1 | N1 | M0 | 1 | 991 | 1 | 859 |
| TCGA-B6-A0IJ | 4.95994630901834 | T3 | N0 | M0 | 0 | 7106 | 0 | 7106 |
| TCGA-B6-A0IQ | 4.58212240679874 | T3 | N1 | M0 | 0 | 4285 | 0 | 4285 |
| TCGA-B6-A0RT | 5.00991593205905 | T3 | N1 | M0 | 0 | 2721 | 0 | 2721 |
| TCGA-B6-A0RU | 5.50733480696659 | T1 | N0 | M0 | 0 | 8605 | 1 | 3076 |
| TCGA-B6-A0WX | 5.24017310624063 | T3 | N1 | M0 | 1 | 639 | 1 | 563 |
| TCGA-B6-A0X1 | 5.59476648991327 | T2 | N1 | M1 | 1 | 7455 | 1 | 490 |
| TCGA-B6-A1KF | 5.95157380791551 | T2 | N1 | M0 | 0 | 3088 | 0 | 3088 |
| TCGA-BH-A0AV | 5.43077701701738 | T1 | N0 | M0 | 0 | 1820 | 0 | 1820 |
| TCGA-BH-A0B3 | 4.76780308526070 | T2 | N1 | M0 | 0 | 1203 | 0 | 1203 |
| TCGA-BH-A0BG | 4.62459808825731 | T1 | N0 | M0 | 0 | 1871 | 0 | 1871 |
| TCGA-BH-A0BL | 5.05245067422951 | T1 | N0 | M0 | 0 | 2278 | 0 | 2278 |
| TCGA-BH-A0BW | 6.02440612127535 | T1 | N0 | M0 | 0 | 2371 | 0 | 2371 |
| TCGA-BH-A0DL | 5.11960518231157 | T2 | N0 | M0 | 0 | 2381 | 0 | 2381 |
| TCGA-BH-A0E0 | 3.26064296121011 | T3 | N3 | M0 | 0 | 134 | 0 | 134 |
| TCGA-BH-A0E6 | 4.28244721863092 | T1 | N0 | M0 | 0 | 293 | 0 | 293 |
| TCGA-BH-A0RX | 5.09820898654248 | T2 | N0 | M0 | 0 | 170 | 0 | 170 |
| TCGA-BH-A0WA | 5.62153323484780 | T1 | N0 | M0 | 0 | 701 | 0 | 701 |
| TCGA-BH-A18G | 4.89493414409595 | T1 | N0 | M0 | 0 | 149 | 0 | 149 |
| TCGA-BH-A18Q | 4.85686122831977 | T2 | N1 | M0 | 1 | 1692 | 0 | 1692 |
| TCGA-BH-A18T | 5.00770232946861 | T2 | N0 | M0 | 1 | 224 | 0 | 224 |
| TCGA-BH-A18V | 5.64342331632819 | T2 | N1 | M0 | 1 | 1556 | 0 | 1556 |
| TCGA-BH-A1F0 | 5.78896493172701 | T1 | N1 | M0 | 1 | 785 | 0 | 785 |
| TCGA-BH-A1FC | 5.52958672377619 | T1 | N1 | M0 | 1 | 3472 | 0 | 3472 |
| TCGA-C8-A12V | 5.19146000346594 | T2 | N0 | M0 | 0 | 385 | 0 | 385 |
| TCGA-C8-A131 | 5.22568753224393 | T2 | N2 | M0 | 0 | 411 | 0 | 411 |
| TCGA-C8-A134 | 4.89739049678354 | T2 | N0 | M0 | 0 | 383 | 0 | 383 |
| TCGA-C8-A27B | 5.53125980110229 | T3 | N0 | M0 | 0 | 439 | 0 | 439 |
| TCGA-D8-A142 | 5.07699512126353 | T3 | N0 | M0 | 0 | 425 | 0 | 425 |
| TCGA-D8-A143 | 5.06411975239010 | T2 | N0 | M0 | 0 | 431 | 0 | 431 |
| TCGA-D8-A147 | 4.95241278194581 | T2 | N0 | M0 | 0 | 584 | 0 | 584 |
| TCGA-D8-A1JL | 5.59204136161724 | T2 | N0 | M0 | 0 | 611 | 0 | 611 |
| TCGA-D8-A1XQ | 4.82478312506360 | T2 | N0 | M0 | 0 | 499 | 0 | 499 |
| TCGA-D8-A27F | 4.66436905866654 | T2 | N0 | M0 | 0 | 488 | 0 | 488 |
| TCGA-D8-A27H | 5.18657978959887 | T2 | N0 | M0 | 0 | 397 | 0 | 397 |
| TCGA-D8-A27M | 5.45762055586349 | T1 | N0 | M0 | 0 | 410 | 0 | 410 |
| TCGA-E2-A14N | 5.26945586600174 | T2 | N1 | M0 | 0 | 1434 | 0 | 1434 |
| TCGA-E2-A14X | 4.09992635055603 | T2 | N2 | M0 | 0 | 972 | 0 | 972 |
| TCGA-E2-A14Y | 4.94316650306365 | T2 | N0 | M0 | 0 | 2109 | 0 | 2109 |
| TCGA-E2-A150 | 5.07090448413358 | T2 | N0 | M0 | 0 | 1935 | 0 | 1935 |
| TCGA-E2-A158 | 4.86511709458666 | T1 | N1 | M0 | 0 | 450 | 0 | 450 |
| TCGA-E2-A159 | 4.87497888938537 | T2 | N0 | M0 | 0 | 762 | 0 | 762 |
| TCGA-E2-A1AZ | 4.89401253905040 | T2 | N1 | M0 | 0 | 2329 | 0 | 2329 |
| TCGA-E2-A1II | 5.12307847150226 | T1 | N0 | M0 | 0 | 1025 | 0 | 1025 |
| TCGA-E2-A1LG | 4.30125839363506 | T2 | N0 | M0 | 0 | 1523 | 0 | 1523 |
| TCGA-E2-A1LH | 4.09927836999171 | T1 | N0 | M0 | 0 | 3247 | 0 | 3247 |
| TCGA-E2-A1LI | 4.67607554776413 | T2 | N1 | M0 | 0 | 3121 | 0 | 3121 |
| TCGA-E2-A1LK | 5.55346188166503 | T4 | N3 | M0 | 1 | 266 | 1 | 155 |
| TCGA-E2-A1LS | 3.46057220826862 | T1 | N0 | M0 | 0 | 1604 | 0 | 1604 |
| TCGA-E9-A1N8 | 5.32150965264845 | T2 | N0 | M0 | 0 | 1039 | 0 | 1039 |
| TCGA-E9-A1N9 | 5.12343444619145 | T2 | N0 | M0 | 0 | 1101 | 0 | 1101 |
| TCGA-E9-A1ND | 5.12278037861605 | T2 | N1 | M0 | 0 | 1266 | 0 | 1266 |
| TCGA-E9-A22G | 5.02727792824224 | T2 | N0 | M0 | 0 | 1239 | 0 | 1239 |
| TCGA-E9-A243 | 4.92693876274409 | T2 | N0 | M0 | 0 | 612 | 1 | 305 |
| TCGA-EW-A1P4 | 4.87088251717843 | T2 | N0 | M0 | 0 | 907 | 0 | 907 |
| TCGA-EW-A1PH | 5.27377695360052 | T1 | N1 | M0 | 0 | 607 | 0 | 607 |
| TCGA-GM-A2DF | 3.82033216083392 | T1 | N1 | M0 | 0 | 2155 | 0 | 2155 |
| TCGA-HN-A2NL | 4.54612893410425 | T2 | N0 | M0 | 0 | 79 | 0 | 79 |
